# Enantiomer signature and carbon isotope evidence for the migration and transformation of DDTs in arable soils across China

**DOI:** 10.1038/srep38475

**Published:** 2016-12-06

**Authors:** Lili Niu, Chao Xu, Siyu Zhu, Huiming Bao, Yang Xu, Hongyi Li, Zhijian Zhang, Xichang Zhang, Jiguo Qiu, Weiping Liu

**Affiliations:** 1 International Joint Research Center for Persistent Toxic Substances (IJRC-PTS), MOE Key Laboratory of Environmental Remediation and Ecosystem Health, College of Environmental and Resource Sciences, Zhejiang University, Hangzhou 310058, China; 2 College of Environment, Zhejiang University of Technology, Hangzhou 310032, China; 3 Department of Geology & Geophysics, Louisiana State University, Baton Rouge, LA, 70803-4101, USA

## Abstract

Due to the adverse impact of DDTs on ecosystems and humans, a full fate assessment deems a comprehensive study on their occurrence in soils over a large region. Through a sampling campaign across China, we measured the concentrations, enantiomeric fractions (EFs), compound-specific carbon isotope composition of DDT and its metabolites, and the microbial community in related arable soils. The geographically total DDT concentrations are higher in eastern than western China. The EFs and δ^13^C of *o,p’*-DDT in soils from western China show smaller deviations from those of racemic/standard compound, indicating the DDT residues there mainly result from atmospheric transport. However, the sources of DDT in eastern China are mainly from historic application of technical DDTs and dicofol. The inverse dependence of *o,p’*-DDT and *p,p’*-DDE on temperature evidences the transformation of parent DDT to its metabolites. Initial usage, abiotic parameters and microbial communities are found to be the main factors influencing the migration and transformation of DDT isomers and their metabolites in soils. In addition, a prediction equation of DDT concentrations in soils based on stepwise multiple regression analysis is developed. Results from this study offer insights into the migration and transformation pathways of DDTs in Chinese arable soils, which will allow data-based risk assessment on their use.

Dichlorodiphenyltrichloroethanes (DDTs) were widely used in the world as effective insecticides for vector-borne diseases control and for crop protection^1^. Due to their detrimental effects to wild life and human health, DDTs were banned decades ago by many countries, including China in 1983. In 2001, they were listed as one of the 12 persistent organic pollutants (POPs) by the Stockholm Convention^2^. In light of the current outbreak of Zika virus infections and their damaging consequences (e.g. microcephaly), an informed debate on whether DDT should be brought back is possible, but only if their post-application environmental processes are well understood.

Historical applications of technical DDTs and dicofol, which usually contains high levels of DDT compounds as impurities in the production process, are the two main sources of DDTs in the environment. DDTs can migrate from soils or transform to their metabolites via various mechanisms, including degradation, volatilization, leaching, run-off, adsorption and plant removal^3^. Geographic locations, meteorological conditions, and soil physicochemical or biological properties are the main factors influencing the environmental behaviors of organic pollutants^4^,^5^. Even though technical DDTs and dicofol were produced and applied more extensively in developed eastern than western China^6^, DDT residues were still found in western China due to their long-range migration as previous studies reported^7^. Like many chiral agricultural pesticides, *o,p’*-DDT consists of one pair of enantiomers. The enantiomeric fraction (EF) can be changed during biodegradation and is a good indicator of past *versus* fresh chiral pollutant inputs^8^,^9^. Compound-specific stable isotope analysis (CSIA) has been used in laboratory and field studies to characterize *in situ* biodegradation processes of organic pollutants^10^,^11^. Due to the different extent of transformation during atmospheric transport^12^,^13^, the EFs and carbon stable isotope compositions of DDT may be totally different between eastern and western part of China. A combination of EF analysis and CSIA might offer additional insights into the migration and transformation of organic contaminants^11^,^14^,^15^, especially on a large scale. After 30 years of ban, the main degradation production of DDTs was found to be DDE in Chinese arable soils^7^. Because of their different sources, DDT and DDE may exhibit distinct isotope characteristics. However, to date, little data from EF analysis and CSIA was obtained on the global cycling and fate of DDT and its metabolites using field samples on a large scale. The study exploring the biological factors influencing the preferential degradation of chiral *o,p’*-DDT is also rather limited, especially at a species level of bacteria. It is necessary to well understand their environmental behaviors under real complicated environment using the techniques mentioned above.

Therefore, in this study, we conducted a nationwide farmland sampling campaign across China and measured the concentrations, EFs and carbon isotope compositions (δ^13^C) of DDT and its main metabolites in arable soils. High-throughput techniques and network analysis were employed to characterize the microbial communities and explore their co-occurrence patterns with DDT and its metabolites in soils. The obtained data were used to estimate the migration and transformation of DDTs on a large scale and to explore the underlying influencing physicochemical and microbiological factors in arable soils after 30 years of DDT ban in China. Furthermore, results from this study would provide basic scientific data for the contamination management and risk avoidance of DDTs in Chinese soils.

## Results

### Concentrations and profiles of DDTs in arable soils across China

**Figure 1:**
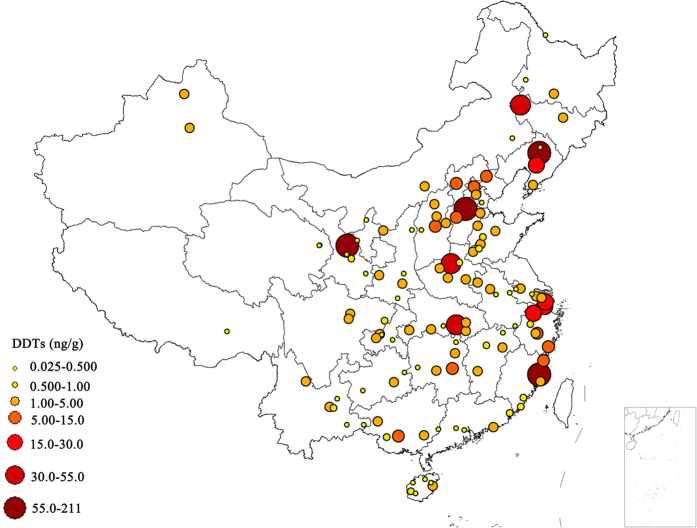
Residue distributions of total DDTs in agricultural soils across China. The map was created using ArcGIS 9.3 software (ESRI, Redlands, California, USA, http://www.esri.com/software/arcgis/arcgis-for-desktop). *Scientific Reports* remains neutral with regard to jurisdictional claims in published maps.

**Figure 2:**
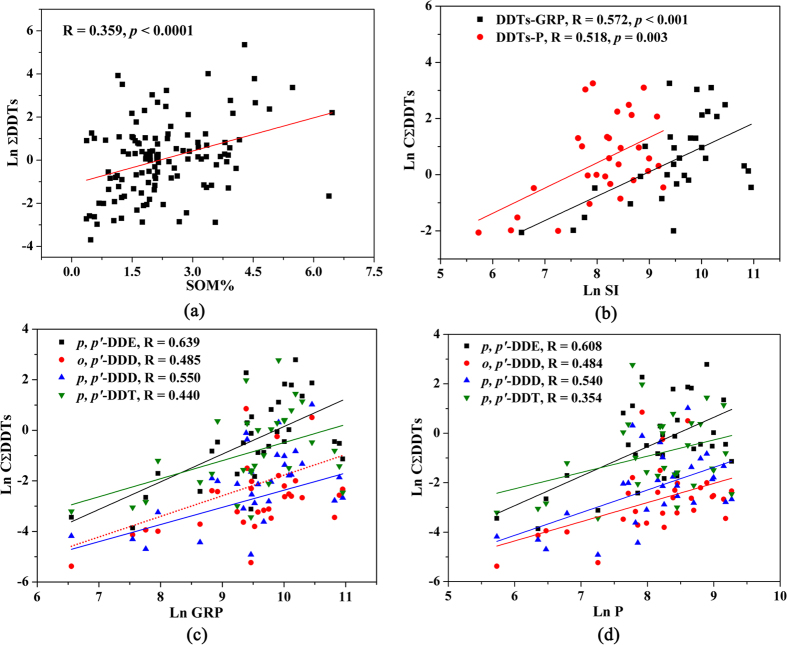
Relationships of DDTs in soils with soil organic matter (SOM) and socioeconomic indicators. GRP: Gross Regional Product; P: Population; SI: Socioeconomic Index.

DDT and its metabolites were detected in all the soil samples we analyzed. Their concentrations are displayed in Table 1. The concentrations of ΣDDTs (sum of *o,p’*-DDE, *p,p’*-DDE, *o,p’*-DDD, *p,p’*-DDD, *o,p’*-DDT and *p,p’*-DDT) ranged from 0.025 to 211 ng/g, with a mean value of 8.06 ng/g. Among the six DDT components, *p,p’*-DDE, which is the main metabolite of DDT, had the highest mean concentration (3.29 ng/g). Regional variation of DDT concentrations and compositions in arable soils across China was mapped in Fig. 1 and Supplementary Fig. 1. Severe DDT contamination was found in soils in eastern China, especially at sites in Shanghai City, and Zhejiang, Fujian, Hebei, Gansu, Liaoning and Jilin provinces. The residue concentrations of DDTs were much lower in western China. The pH and soil organic matter (OM) content ranged from 4.01 to 8.70 and 0.367 to 6.88%, respectively^16^. A significant but weak correlation between ∑DDT concentrations and OM (R = 0.359, *p* < 0.0001, Fig. 2a) was found in this study. In addition, a significant relationship (*p* < 0.05) was also identified between the residual levels of DDTs and socioeconomic indicators (SIs), including gross regional product (GPR) and population (P) (Fig. 2b and c)^17^. Among the DDT components, *p,p’*-DDE was the most abundant in 63.1% of the samples, especially in soils from eastern and northern China (Fig. 1b). *p,p’*-DDT, the main component in technical DDTs, is the dominant component in 30.3% of soil samples located mainly at sites in Shanghai City, and in Gansu, Shanxi, Fujian and Hunan provinces.

Considering complex factors impacting on the residue levels of DDTs, stepwise multiple regression analysis was employed to explore the dominant factors and develop a prediction model. Based on the results of correlation analysis above, variables including organic matter, temperature, longitude and latitude were included. However, after significance and multicollinearity testing, temperature was excluded in the analysis. The significant prediction equation is as follows:


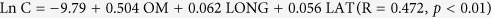


where, C is the residue concentrations of ∑DDTs in agricultural soils (ng/g); OM is the content of organic matter (%); LONG is the longitude of sampling site (°E); LAT is the latitude of sampling site (°N).

Technical DDTs are composed of 75% *p,p’*-DDT, 15% *o,p’*-DDT, 5% *p,p’*-DDE, <0.5% *p,p’*-DDD, <0.5% *o,p’*-DDD, <0.5% *o,p’*-DDE and <0.5% unidentified compounds^18^. In this study, we found more than half of the analyzed soils have the ratio of (*p,p’*-DDE+ *p,p’*-DDD)/*p,p’*-DDT > 1 and of *o,p’*-DDT/*p,p’*-DDT < 0.2 (Supplementary Fig. 2a). The ratio of (*p,p’*-DDE+ *p,p’*-DDD)/*p,p’*-DDT was much higher in soils with higher organic matter contents (>2.3%) and higher temperatures (>14 °C) (Supplementary Fig. 2b and c). In addition, DDD/DDE ratios showed a weak positive correlation with elevation of the sampling sites (Supplementary Fig. 2d).

### Enantiomeric fraction of *o,p’*-DDT

**Figure 3:**
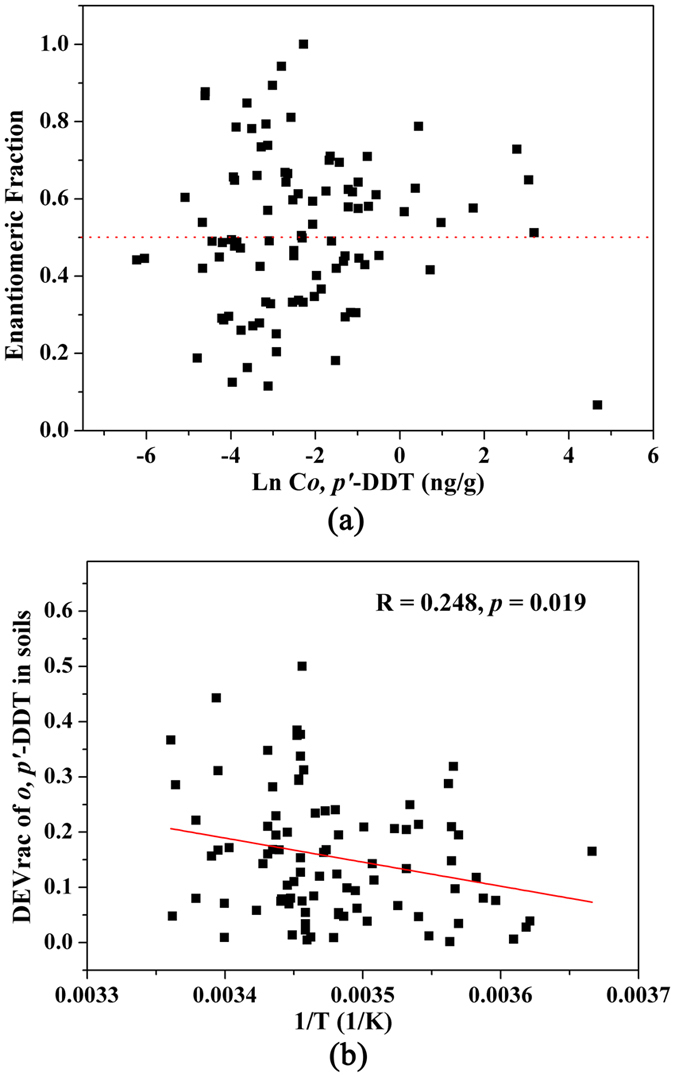
Enantiomeric signatures of *o,p’*-DDT in soils (**a**) and the relationship between DEVrac (deviation from racemic compound) of *o,p’*-DDT and temperature (**b**).

Ninety-four soil samples with sufficient *o,p’*-DDT levels were selected for enantiomeric analysis. In general, the EF values of *o,p’*-DDT in soils varied greatly, ranging from 0.066 to 1(Fig. 3a). The EF values deviating from 0.5 were observed in most soil samples. There were 51.1% of the samples having EF values lower than 0.5. Geographically, the EFs of *o,p’*-DDT were below 0.5 in over half of the sampled soils in eastern and northeastern China, while above 0.5 in more than 50% soils in central and western China (Supplementary Table 1). The deviation from racemic (DEVrac = absolute values of 0.5 − EF) is a parameter measuring the degree of enantioselective metabolism regardless of which enantiomer is preferentially degraded^9^. The DEVrac of *o,p’*-DDT was found to slightly increase with temperature (Fig. 3b). However, the soil pH was not found to well correlate with the concentration of either DDTs or enantiomer residues like those reported in Zhang *et al*.^19^.

### Compound-specific carbon isotope composition of *o,p’*-DDT and *p,p’*-DDE

**Figure 4:**
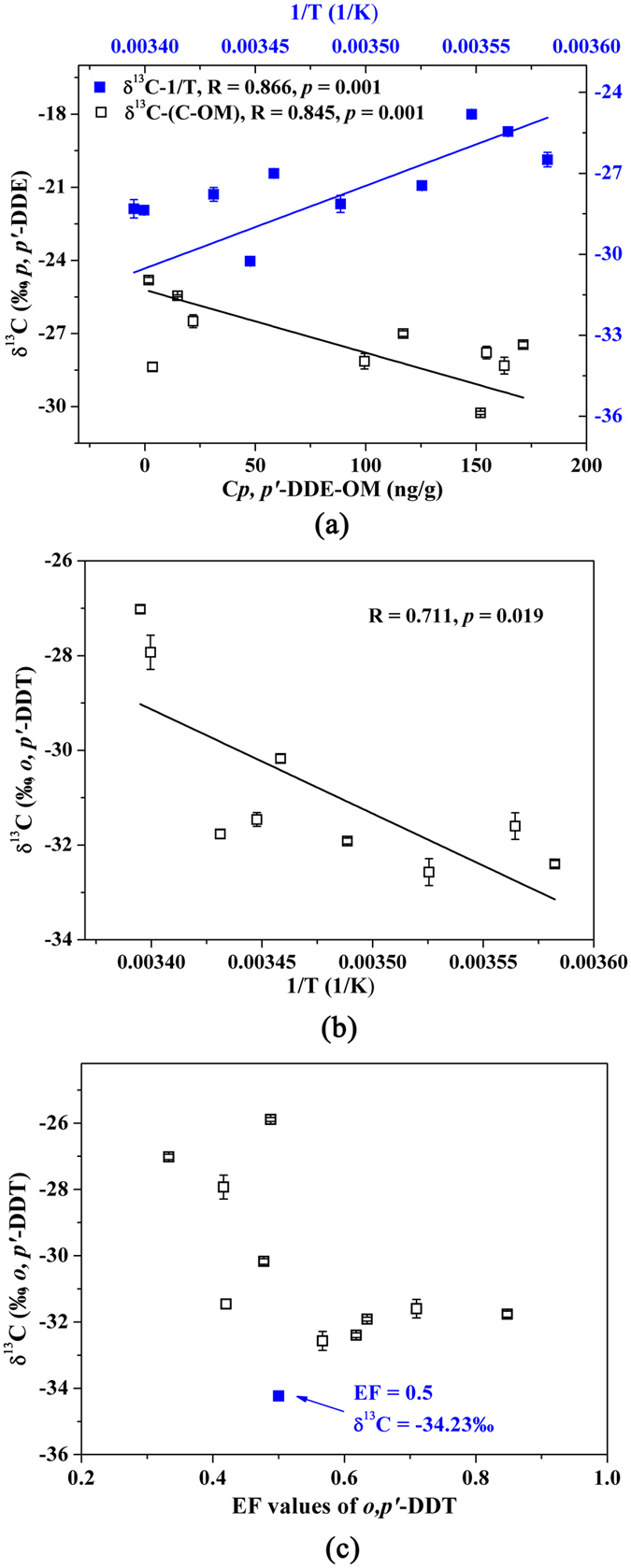
Stable carbon isotope signatures of *p,p’*-DDE (**a**) and *o,p’*-DDT (**b**) and the relationships with enantiomeric fraction of *o,p’*-DDT (**c**) in soils.

Compound-specific stable isotope analysis has been recognized in contemporary environmental science and successfully used in some studies as an indicator to provide insight into the sources and *in situ* biodegradation processes of pollutants^10^,^11^,^14^. Therefore, in this study, 10 representative soil samples were selected for stable carbon isotope analysis according to geographic locations, sources, and concentration ranges of DDTs (Supplementary Table 2). The carbon isotope composition of technical DDTs and dicofol may be different by various manufactures and even by produced dates^20^. In this study, we assumed the δ^13^C of DDTs obtained from Dr. Ehrenstorfer GmbH (Augsburg, Germany) as the initial stable carbon isotope composition of DDTs applied for agriculture. The δ^13^C of *p,p*’-DDE ranged from −24.81 ± 0.12‰ to −30.26 ± 0.06‰ and deviated either positively or negatively from those of the initial product (−28.77 ± 0.06‰, Supplementary Table 2), with a negative dependence on the normalized concentration by OM (R = 0.845, Fig. 4a). The δ^13^C of *o,p*’-DDT ranged from −25.89 ± 0.06‰ to −32.57 ± 0.28‰, deviating only positively from the δ^13^C of the initial product (−34.23 ± 0.13‰) but with a wider range (Supplementary Table 2). In addition, the δ^13^C of *p,p’*-DDE correlated negatively with the mean annual temperature of each site (R = −0.868, Fig. 4a), while those of *o,p’*-DDT correlated positively with temperature (R = 0.711, Fig. 4b). The stable carbon isotope and enantiomer compositions of *o,p’*-DDT are jointly displayed in Fig. 4c for comparison. The δ^13^C and EF values both deviated from the racemic standard product. More positive values of δ^13^C were observed in samples with EF < 0.5 than those with EF > 0.5.

### Microbial communities and DDTs

**Figure 5:**
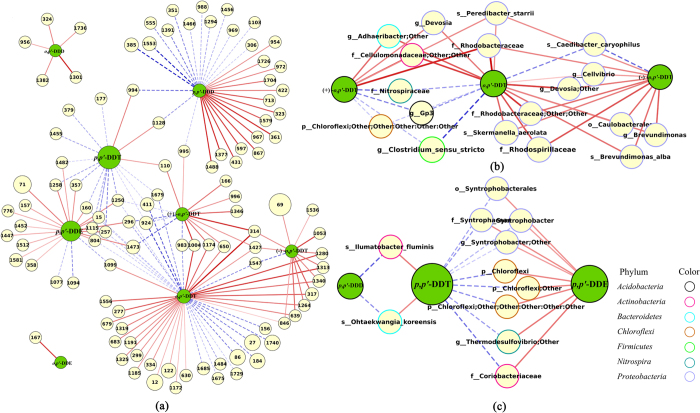
Mutual influences between microbial community and DDT residues in arable soils across China based on the Network Analysis. The nodes with number were the bacterial taxa and their names were listed in Supplementary Information Table 3. The blue and red edges represent negative and positive correlations, respectively, and the stronger is in correlation, the darker in color.

Nineteen bacterial phyla were identified via Illumina sequencing from 28 samples across China. *Proteobacteria* was the most abundant phylum, followed by *Actinobacteria* (Supplementary Fig. 3). Network analyses showed that *Acidimicrobiales* and *Gp3* correlated negatively and positively, respectively, with OM. Considerable numbers of taxa were observed to correlate well with the fractions of *p,p’*-DDT, *o,p’*-DDT, *p,p’*-DDE and *p,p’*-DDD, while few with the fractions of *o,p’*-DDE and *o,p’*-DDD (*p* < 0.01) (Fig. 5 and Supplementary Table 3). Bacteria of *Thermodesulfovibrio*, *Coriobacteriaceae*, *Syntrophobacterales* and unknown species of *Chloroflexi* correlated negatively with the abundance of *p,p’*-DDT, while positively with *p,p’*-DDE. Three species, including *lamia_majanohamensis*, *ilumatobacter_fluminis* and *Ohtaekwangia_koreensis*, showed inverse correlations with the relative abundance of parent compound *p,p’*-DDT and metabolite *p,p’*-DDD. Fifty-seven taxa of 9 phyla were found to strongly correlate with the fractions of *o,p’*-DDT or its enantiomers (Fig. 5a). Strong negative correlations was found between the abundance of *Coribacteriaceae, Syntrophobacterales* and *Chloroflexi* with *o,p’-*DDT. *Peredibacter_starrii* and *Rhodobacteraceae* correlated positively with both enantiomers of *o,p’*-DDT. In addition, both (+)-*o,p’*-DDT and *o,p’*-DDT showed positive correlations with *Devosia*, *Adhaeribacter* and *Cellulomonadaceae*, but negative correlations with *Gp3*, *Clostridium_sensu_stricto*, *Nitrospiraceae* and *Chloroflexi*. The abundance of both *o,p’*-DDT and its (−)-enantiomer has negative correlations with *Caedibacter_caryophilus* and positive correlations with *Skermanella_aerolata*, *Brevundimonas_alba*, *Cellvibrio*, *Brevundimonas*, *Devosia*, *Rhodobacteraceae*, and *Caulobacterales*.

## Discussion

Residues of DDTs are found to be ubiquitous in agricultural soils across China even after 30-year ban of their application. Being the main metabolite of *p,p’*-DDT, *p,p’*-DDE is more persistent than other DDT components^21^. That explains its most abundant concentrations in soils. Compared to recent worldwide data from agricultural soils (Supplementary Table 4), the ∑DDT concentrations measured in the present study are relatively low. The ban of technical DDT usage since 1983 and the decreasing production of dicofol may be responsible for the decline. The lower DDT concentrations observed in this study may also be the result of dissipation of DDTs in soils over years.

Historical usage is a crucial factor influencing the DDT concentrations in the environment. In this study, the spatial distribution pattern of ∑DDTs in arable soils roughly overlaps with the usage inventory map of technical DDTs in 1951–1983 and dicofol in 1984–2003 in China^22^. The average ratio of *o,p’*-DDT/*p,p’*-DDT in technical DDTs is 0.19, which is much lower than that in dicofol (7.0)^23^. The compositions of DDTs can be altered by biological and abiotic degradation, volatilization, and re-deposition in the environment due to their different properties. Therefore, the change in the ratios of different DDT compounds can be employed to gauge their age and origin^19^. The ratio data show that the residues of DDTs are mainly from historical technical DDTs and secondarily from the later dicofol usage. However, the increasing abundance of *p,p’*-DDT component with time compared to our previous study^7^ also reveals an increased fresh input of DDTs, which might be from the use of technical DDTs. Enantioselective degradation could result in enantiomer fractionation of chiral pollutants^9^,^12^. As our data show, the EF values of *o,p’*-DDT varied greatly in arable soils across China. Similar to previous studies^9^,^19^,^24^, most samples showed EFs deviating from the racemic compound, suggesting aged DDTs are dominating in today’s soils. Less amount of DDTs were applied in western China, where residual DDTs were still found. The EF values of *o,p’*-DDT in western China also deviated from that of the racemic compound. This might be due to long-range transport from other places and subsequent wet or dry deposition. The enantiomeric signatures of chiral chemicals may retain racemic or nearly racemic in the air^12^. Therefore, their smaller DEVrac of *o,p’*-DDT (mean = 0.016) compared to those at other sites further supports a significant DDT source from atmospheric deposition in western China. In addition, the different physiochemical and biological features of the soil environments might also contribute to the different EF values among the four geographic regions across China. Other studies also obtained EF values of *o,p’*-DDT either > or <0.5, indicating a complex degradation preference^9^,^19^,^24^. It has been reported that the enantioselective degradation of chiral pesticides can be altered by soil pH, OM and redox conditions^19^,^25^. In addition, environmental changes, such as tropical deforestation and global warming could also lead to enantiomer preferences^26^. In this study, we did find a significant enantioselective degradation of *o,p’*-DDT, but not on the enantiomer preference. It implies that the preferential degradation of *o,p’*-DDT enantiomers may be modulated by the integrated effects of multiple environmental factors in soil environment. However, the absolute degree of the enantioselective degradation of *o,p’*-DDT was found to be slightly elevated by temperature. It might be due to the higher enantioselective degradation rates related to higher microbial activities in sites with higher temperature.

Compound-specific isotope compositions can offer insights into the pathways of *in situ* degradation of POPs in various environments^10^. Badea *et al*.^14^ recommended a δ^13^C difference of at least 2‰ for reliably interpreting the sources of different sites considering environmental complexity. Different δ^13^C of *p,p*’-DDE and *o,p*’-DDT were observed in different soils in this study (Fig. 4 and Supplementary Table 2). *o,p’*-DDT is the main contributor of dicofol-type DDT pollution. The δ^13^C of *o,p*’-DDT in all measured soils are higher than that of the possible starting DDT. It indicates a Rayleigh-style degradation process which preferentially degrades ^12^C-enriched DDTs and results in the δ^13^C increase in residual DDTs in soils. However, *p,p’*-DDE is the main metabolite and more persistent than parent DDT^4^. Its carbon isotope composition could be influenced by both the degradation from parent DDTs (^12^C-enriched process) and its own dissipation (^13^C-enriched process). The fact that the Shanghai soil with an abnormally high concentration of *p,p’*-DDT (15.9 ng/g) bearing the lowest δ^13^C of *p,p’*-DDE is consistent with this explanation (Supplementary Table 2). The positive correlation between mean annual temperature and the δ^13^C of *o,p’*-DDT appears robust, a pattern we hypothesize as the consequence of warmer regions having higher degradation rates and subsequent higher ^13^C enrichment than cooler regions. Interestingly, this pattern seems to contradict to that of the main metabolite *p,p*’-DDE. It is likely due to a larger degree of degradation on *p,p’*-DDT than on *p,p’*-DDE, resulting in more ^12^C enrichment in *p,p’*-DDE at warmer sites. This trend is in agreement with the lower and higher fractions of *p,p’*-DDT and *p,p’*-DDE, respectively, at the sites with higher temperature (eastern China). In addition, DDE residues in eastern China are mainly from the degradation of DDT. Their residues in colder western China, where less amount of DDTs were used than eastern China, are mostly from the long-range transport via air from eastern China^7^. It is known that carbon isotope fractionation is smaller during physical than biodegradation process^13^. Therefore, more positive δ^13^C is found for *p,p’*-DDE in western China, which could partly account for the negative correlation between its δ^13^C and local temperature. This interpretation is also suitable for the positive correlation between the δ^13^C of *o,p’*-DDT and temperature. All these hypotheses should be further tested with controlled experiments or additional sampling. Milosevic *et al*.^11^ found more positive δ^13^C of BTEX at contaminated landfill with lower concentrations, which was due to their microbial degradation and subsequent enrichment of ^13^C. Likewise, this trend was also observed for *p,p’*-DDE in this study. However, the possible reason for our findings might be different due to the different initial usage amounts of DDTs across China. More technical DDTs and dicofol have been applied in eastern China than western China. Therefore, the relative newer and higher concentrations of DDT residues might be the main reason for the more negative δ^13^C of *p,p’*-DDE in eastern China.

The combination of the enantiomer and stable carbon isotope fractionation, in a two-dimensional approach, may aid to further understand the degradation pathways of chiral *o,p’*-DDT. Both the EF and δ^13^C values of *o,p’*-DDT in the measured samples differentiated from those of racemic standard products, further suggesting active metabolism of DDTs in soils. Interestingly, the stable carbon isotope compositions of *o,p’*-DDT showed different characteristics in soils with EF > 0.5 or <0.5. The δ^13^C of *o,p’*-DDT in soils with EF > 0.5 displayed more negative values than those in soils with EF < 0.5. Bashir *et al*. observed higher stable carbon isotope fractionation for (+)- than (−)-*α*-HCH during biodegradation^27^. If the enantiomers of *o,p’*-DDT also have the similar carbon isotope fractionation, the observed different isotope fractionation patterns between EF > 0.5 (preferential degradation of (−)-enantiomer) and <0.5 (preferential degradation of (+)-enantiomer) could be explained. This prediction can be further tested using enantiomer-specifics table isotope analysis in future laboratory study.

The initial use of chemicals can lead to a primary distribution pattern, which is controlled by the usage amount of chemicals^28^. A strong association between DDTs and SIs, similar to that reported by Simonich *et al*.^29^, was found in the present study. It is consistent with the fact that accumulatively more DDTs were produced and applied in more economically developed regions in China (eastern China). Once chemicals are released, their volatilization, deposition and runoff, etc., may lead them disperse in the environment and form the secondary distribution pattern^28^. The redistribution of organic pollutants depends on various environmental conditions and soil physicochemical or biological properties^9^,^16^,^17^7,^19^. Temperature is an important factor influencing the degradation of DDTs. In arable soil sites with higher temperatures, the degradation of DDTs is enhanced and predominated by the aerobic metabolism of DDT to DDE. Soil OM has been reported to have the ability to sequester environmental contaminants in soils and a linear correlation between OM and POP residue concentrations has been obtained in an equilibrated soil-air system^30^. A typical secondary distribution characteristics modulated by soil OM were found in soils from south-western Spain and Zhejiang, China^19^,^31^. However, we only observed a weak correlation between OM and the residue concentration of DDTs in soils. This is expected because of the complexity of factors impacting the residue concentration of soil DDTs and the nature of our large-scale sampling. In addition, the enhanced metabolism of *p,p’*-DDT to *p,p’*-DDE and *p,’p*-DDD by soil OM and temperature was observed in this study. It might be associated with the higher microbial activities in sites with higher soil OM and temperature. The R^2^ value of the prediction equation based on stepwise multiple regression analysis is 0.222. It means that 22% of the total variations in DDT concentrations are explained by OM, longitude and latitude. The content of OM is the principal factor among these variables. Although the prediction model is significantly developed, the R^2^ value is still a little small. The initial usage amounts of technical DDTs might be the most predominant factor influencing the distribution and residue levels of DDTs in agricultural soils across China.

Soil microbial communities, which are influenced by environmental conditions, play critical roles in the degradation of contaminants. Various bacteria, such as *Serratia marcescens* DT-1P, *Pseudomonas fluorescens* and *Alcaligenes* sp. KK, *Sphingobacterium* sp. D6 and *Chryseobacterium* sp. PYR2 and *Bacillus* sp. BHD-4, have been documented to be capable of converting DDTs to their metabolites^32^,^33^,^34^,^35^,^36^,^37^. However, the abundances of these bacterial strains were not found to correlate directly with those of DDT residues in the present study. It might be due to the very low abundance of these bacteria in soils and/or the complex environmental conditions inherited in a field study. Microbes, including *Chloroflexi*, *Coriobacteriaceae* (belong to *Actinobacteria*) and *Syntrophobacter*, *Thermodesulfovibrio*, *Caedibacter_caryophilus* (belong to *Gammaproteobacteria*), whose abundances correlate negatively with the fraction of *p,p’*-DDT and positively with that of *p,p’*-DDE, may be capable of biodegrading DDTs. The significant positive correlation between the degree of DDT degradation and the abundance of *Actinobacteria* and *Gammaproteobacteria* was also identified by Sun *et al*.^38^. Sulfate- and iron- reducing bacteria distribute widely in the environment. They have been reported to be capable of anaerobically dechlorinating organochlorine chemicals, including DDTs, via co-metabolism with iron or sulfate reduction^39^,^40^,^41^. In this study, some sulfate- and iron reducing bacteria (*Syntrophobacte*r and *Thermodesulfovibrio*, Fig. 5b) were found to correlate negatively with the abundance of *p,p’*-DDT but positively with that of its aerobic metabolite *p,p’*-DDE. It suggests that these bacteria may have the potency to dechlorinate DDT to DDE in aerobic environments. *Clostridia* are obligate anaerobes and found to be affected by the pollution of metal and petroleum hydrocarbons^42^,^43^, and may thrive in *o,p’*-DDD-polluted soil across China as suggested by Guo *et al*.^44^. In addition, the similarly positive correlations between *o,p’*-DDT with *Alphaproteobacteria* (including *Devosia*, *Brevundimonas_alba*, *Skermanella_aerolata* and *Caulobacterales* found in this study) and *Sphingobacteria (Adhaeribacter* found in this study) were also observed in Guo *et al*.^44^ For chiral chemicals, their enantioselective degradation is mainly the result of microbial activities but not abiotic reactions^26^,^45^. The enantiomeric fractionation of chiral pollutants could be caused by different degradation rates of the same or different enzymes^46^. In this study, *Peredibacter_starrii* and *Rhodobacteraceae* correlate positively with both (+)-*o,p’*-DDT and (−)-*o,p’*-DDT, indicating that different degradation rates by the same bacteria may be one possible explanation for the enantioselective dissipation of *o,p’*-DDT in soils. This result is consistent with those reported by Lewis *et al*.^26^. They found the enantiomers of the chiral chemicals they studied were transformed at different rates in all soil samples and bacterial isolates under field and laboratory experiments. Some bacteria, such as *Gp3, Clostridium_sensu_stricto*, *Nitrospiraceae*, *Chloroflexi* and *Caedibacter_caryophilus*, are only capable of degrading (+)- or (−)-enantiomer of *o,p’*-DDT. Thus, different bacteria can degrade different enantiomers which could also result in the EF values deviating from 0.5. Generally, the taxa we found to correlate well with the stereoisomers and metabolites of DDTs in this study are mostly at genus and species level, not of higher levels. Our results demonstrate that DDT residues can influence the specific microbes and they could in turn impact the redistributions of DDTs in Chinese arable soils.

## Methods

### Sample collection

A total of 123 surface soils (0–20 cm) were collected from agricultural fields covered 31 provinces across China in April and May 2013. Detailed information on sampling locations and strategy was described in our previous study and briefly presented in the Supplementary Information (Text and Supplementary Fig. 4).

### Sample extraction and analysis

A mix of 6 DDT compounds (*o,p’*-DDE, *p,p’*-DDE, *o,p’*-DDD, *p,p’*-DDD, *o,p’*-DDT and *p,p’*-DDT) (their molecular structures are listed in Supplementary Fig. 5) was purchased from Dr. Ehrenstorfer GmbH (Augsburg, Germany). Detailed procedures of sample pretreatment were described in the Supplementary Information. Briefly, after being Soxhlet-extracted with dichloromethane (DCM), the concentrated extracts were cleaned up through a column containing anhydrous granular sodium sulfate (Na_2_SO4), neutral silica gel, alumina, and florisil. Then, the target analytes eluted by hexane/DCM (7:3) were further concentrated for analysis.

The concentrations of DDT and its metabolites were measured on an Agilent 7890A gas chromatograph (GC-ECD, Agilent Technologies, Avondale, PA, USA) equipped with BGB-172 chiral capillary column (20% *tert*-butyldimenthylsilylated-*β*-cyclodextrin in OV-1701, 30 m × 0.25 mm × 0.25 μm; BGB Analytik AG, Switzerland). The determination of enantiomeric fractions (EFs) of chiral *o,p’*-DDT were performed by a gas chromatograph-mass spectrometry (Agilent 7890A GC-5975C MS). A larger quantity of soils was used to obtain sufficient amounts of targets for isotope analysis. The stable carbon isotope composition (δ^13^C) of DDT and its metabolites was measured using an Agilent 7890A GC coupled to a GV Isoprime IRMS (GV Instruments, UK) (GC-C-IRMS) via a modified GC5 combustion interface at College of Environmental and Resource Sciences, Zhejiang University. Due to the limitation of concentration and the existence of interfering substances, only the δ^13^C of *p,p’*-DDE and *o,p’*-DDT were determined.

The instrumental conditions and columns used for the separation of DDT isomers in GC-ECD and GC-C-IRMS and the enantiomers in GC-MS were given in detail in the Supplementary Information.

### DNA extraction and Illumina Miseq sequencing

Soil DNA was isolated from a 0.25 g fresh soil sample using Power Soil DNA kit (Mo Bio Laboratories, USA) based on the instructions of the manufacturer. 16S rRNA gene amplicons were pair-end sequenced at Shenzhen Huada Genomics Institute by using Illumina Miseq (300 bp-PE). The detailed procedures were described in Supplementary Information. The sequencing data were all deposited in GenBank (Accession Number: SRP072159).

### Quality control and quality assurance

Blank controls were ministered for every 15 soil samples to check potential cross-contaminations and interferences. The recoveries of DDTs were in the range of 82.8% to 105%. The mean recoveries of surrogates, including TCmX and PCB209 were 93.9 ± 12% and 89.2 ± 15%, respectively. Three times of signal-to-noise ratio were calculated as the limits of detection (LOD), which ranged from 0.003 to 0.005 ng/g for target compounds. Potential thermal degradation of DDTs was checked daily and a ratio less than 6% was satisfied. Duplicate experiments were performed in the whole procedure.

The EF values of *o,p’*-DDT are defined as the portion of the (+)-enantiomer to the sum of the (−)- and (+)-enantiomers. Racemic standard of *o,p’*-DDT with every 10 samples were injected and the mean EF value was 0.498 ± 0.005. Data was deemed acceptable only if the EF differences between the two monitored ions were within 0.05. The *o,p*’-DDT in samples was considered to be racemic when the EF value was within the 95% confidence interval (CI) of the standard.

## Additional Information

**How to cite this article**: Niu, L. *et al*. Enantiomer signature and carbon isotope evidence for the migration and transformation of DDTs in arable soils across China. *Sci. Rep.* **6**, 38475; doi: 10.1038/srep38475 (2016).

**Publisher's note:** Springer Nature remains neutral with regard to jurisdictional claims in published maps and institutional affiliations.

## Supplementary Material

Supplementary Information

## Figures and Tables

**Table 1:** Descriptive Statistical Summary of DDT Component Concentrations in Agricultural Soils across Mainland China (ng/g, soil).

	Percentiles					Mean	Min	Max	SD^a^	CV(%)^b^	DF(%)^c^
	5^th^	25^th^	50^th^	75^th^	95^th^						
*o,p’*-DDE	0.008	0.020	0.035	0.066	0.469	0.137	BDL^d^	5.57	0.539	392	99.2
*p,p’*-DDE	BDL^d^	0.118	0.428	1.355	17.1	3.29	0.008	72.8	9.98	303	100
*o,p’*-DDD	0.005	0.015	0.039	0.105	0.853	0.242	BDL^d^	4.68	0.730	301	98.4
*p,p’*-DDD	0.007	0.019	0.078	0.216	2.73	0.469	BDL^d^	12.9	1.45	309	97.5
*o,p’*-DDT	BDL^d^	0.012	0.045	0.187	1.55	0.711	BDL^d^	24.0	3.24	456	82.8
*p,p’*-DDT	0.009	0.048	0.253	0.783	10.7	3.21	BDL^d^	115	14.4	447	96.7
∑DDTs^e^	0.066	0.313	1.18	2.75	33.5	8.06	0.025	211	27.1	336	100

^a^SD: standard deviation.

^b^CV: coefficient variation.

^c^DF: detection frequency.

^d^BDL: below detection level.

^e^∑DDTs: sum of *o,p’*-DDE, *p,p’*-DDE, *o,p’*-DDD, *p,p’*-DDD, *o,p’*-DDT and *p,p’*-DDT.

## References

[b1] Caraballo H. & King K. Emergency department management of mosquito-borne illness: malaria, dengue, and West Nile virus. Emerg. Med. Pract. 16, 1–23 (2014). 25207355

[b2] Stockholm Convention on Persistent Organic Pollutants. UNEP: persistent organic pollutants. http://www.pops.int/documents/convtext/convtext_en.pdf (2001).

[b3] Singh G., Kathpal T. S., Spencer W. F. & Dhankar J. S. Dissipation of some organochlorine insecticides in cropped and uncropped soil. Environ. Pollut. 70, 219–239 (1991). 15092134 10.1016/0269-7491(91)90011-k

[b4] Scholtz M. T. & Bidleman T. F. Modelling of the long-term fate of pesticide residues in agricultural soils and their surface exchange with the atmosphere: Part II. Projected long-term fate of pesticide residues. Sci. Total Environ. 377, 61–80 (2007). 17346778 10.1016/j.scitotenv.2007.01.084

[b5] Niu L. L. . Status, Influences and risk assessment of hexachlorocyclohexanes in agricultural soils across China. Environ. Sci. Technol. 47, 12140–12147 (2013). 24094369 10.1021/es401630w

[b6] Li Y. F., Cai D. J. & Singh A. Historical DDT use trends in China and usage data gridding with 1/4 by 1/6 longitude/latitude resolution. Adv. Environ. Res. 2, 497–506 (1999).

[b7] Tang M. L. . Assessing the underlying breast cancer risk of Chinese females contributed by dietary intake of residual DDT from agricultural soils. Environ. Int. 73, 208–215 (2014). 25160079 10.1016/j.envint.2014.08.001

[b8] Niu L. L., Xu C., Xu Y., Zhang C. L. & Liu W. P. Hexachlorocyclohexanes in tree bark across Chinese agricultural regions: spatial Distribution and enantiomeric signatures. Environ. Sci. Technol. 48, 12031–12038 (2014). 25252210 10.1021/es503372g

[b9] Kurt-Karakus P. B., Bidleman T. F. & Jones K. C. Chiral organochlorine pesticide signatures in global background soils. Environ. Sci. Technol. 39, 8671–8677 (2005). 16323761 10.1021/es051004c

[b10] Zhang N. . Compound specific stable isotope analysis (CSIA) to characterize transformation mechanisms of *α*-hexachlorocyclohexane. J. Hazard. Mater. 280, 750–757 (2014). 25238192 10.1016/j.jhazmat.2014.08.046

[b11] Milosevic N. . Combined isotope and enantiomer analysis to assess the fate of phenoxy acids in a heterogeneous geologic setting at an old landfill. Water Res. 47, 637–649 (2013). 23168311 10.1016/j.watres.2012.10.029

[b12] Bidleman T. F., Jantunen L. M., Kurt-Karakus P. B. & Wong F. Chiral persistent organic pollutants as tracers of atmospheric sources and fate: review and prospects for investigating climate change influences. Atmos. Pollut. Res. 3, 371–382 (2012).

[b13] Hunkeler D. . Carbon and chlorine isotope ratios of chlorinated ethenes migrating through a thick unsaturated zone of a sandy aquifer. Environ. Sci. Technol. 45, 8247–8253 (2011). 21870853 10.1021/es201415k

[b14] Badea S. L. & Danet A. F. Enantioselective stable isotope analysis (ESIA) - a new concept to evaluate the environmental fate of chiral organic contaminants. Sci. Total Environ. 514, 459–466 (2015). 25687672 10.1016/j.scitotenv.2015.01.082

[b15] Jammer S., Voloshenko A., Gelman F. & Lev O. Chiral and isotope analyses for assessing the degradation of organic contaminants in the environment: Rayleigh dependence. Environ. Sci. Technol. 48, 3310–3318 (2014). 24471759 10.1021/es4039209

[b16] Niu L. L., Xu Y., Xu C., Yun L. X. & Liu W. P. Status of phthalate esters contamination in agricultural soils across China and associated health risks. Environ. Pollut. 195, 16–23 (2014). 25194267 10.1016/j.envpol.2014.08.014

[b17] National Bureau of statistics of China. China Statistical Yearbook. China Statistics Press (2013).

[b18] World Health Organization (WHO). DDT and its derivatives. (New York, 1979).

[b19] Zhang A. P., Chen Z. Y., Ahrens L., Liu W. P. & Li Y. F. Concentrations of DDTs and enantiomeric fractions of chiral DDTs in agricultural soils from Zhejiang Province, China, and correlations with total organic carbon and pH. J. Agric. Food Chem. 60, 8294–8301 (2012). 22867105 10.1021/jf3024547

[b20] Drenzek N. J. . Stable chlorine and carbon isotopic compositions of selected semi-volatile organochlorine compounds. Org. Geochem. 33, 437–444 (2002).

[b21] Boul H. L., Garnham M. L., Hucker D., Baird D. & Aislable J. Influence of agricultural practices on the levels of DDT and its residues in soil. Environ. Sci. Technol. 28, 1397–1402 (1994). 22165920 10.1021/es00057a004

[b22] Wang Q. J., Liu L. Y., Qi H., Liu G. M. & Li Y. F. The pollution status of DDTs in various environmental medium of China. China POPs Forum 2011 & the 6th National Symposium on Persistent Organic Pollutants. (Haerbin, Heilongjiang Province, China 2011) (in Chinese).

[b23] Qiu X. H., Zhu T., Yao B., Hu J. X. & Hu S. W. Contribution of dicofol to the current DDT pollution in China. Environ. Sci. Technol. 39, 4385–4390 (2005). 16047771 10.1021/es050342a

[b24] Li J., Zhang G., Qi S. H., Li X. D. & Peng X. Z. Concentrations, enantiomeric compositions, and sources of HCH, DDT and chlordane in soils from the Pearl River Delta, South China. Sci. Total Environ. 372, 215–224 (2006). 17074382 10.1016/j.scitotenv.2006.09.023

[b25] Buerge I. J., Poiger T., Muller M. D. & Buser H. R. Enantioselective degradation of metalaxyl in soils: Chiral preference changes with soil pH. Environ. Sci. Technol. 37, 2668–2674 (2003). 12854703 10.1021/es0202412

[b26] Lewis D. L. . Influence of environmental changes on degradation of chiral pollutants in soils. Nature 401, 898–901 (1999). 10553905 10.1038/44801

[b27] Bashir S., Fischer A., Nijenhuis I. & Richnow H. H. Enantioselective carbon stable isotope fractionation of hexachlorocyclohexane during aerobic biodegradation by Sphingobium spp. Environ. Sci. Technol. 47, 11432–11439 (2013). 24007541 10.1021/es402197s

[b28] Li Y. F. . Polychlorinated biphenyls in global air and surface soil: distributions, air-soil exchange, and fractionation effect. Environ. Sci. Technol. 44, 2784–2790 (2010). 20384373 10.1021/es901871e

[b29] Simonich S. L. & Hites R. A. Relationships between socioeconomic indicators and concentrations of organochlorine pesticides in tree bark. Environ. Sci. Technol. 31, 999–1003 (1997).

[b30] Borisover M. D. & Graber E. R. Specific interactions of organic compounds with soil organic carbon. Chemosphere 34, 1761–1776 (1997).

[b31] Munoz-Arnanz J. & Jimenez B. New DDT inputs after 30 years of prohibition in Spain. A case study in agricultural soils from south-western Spain. Environ. Pollut. 159, 3640–3646 (2011). 21864957 10.1016/j.envpol.2011.07.027

[b32] Fang H., Dong B., Yan H., Tang F. F. & Yu Y. L. Characterization of a bacterial strain capable of degrading DDT congeners and its use in bioremediation of contaminated soil. J. Hazard. Mater. 184, 281–289 (2010). 20828928 10.1016/j.jhazmat.2010.08.034

[b33] Xie H. . Isolation and degradation ability of the DDT-degrading bacterial strain KK. Environ. Earth Sci. 62, 93–99 (2011).

[b34] Qu J., Xu Y., Ai G. M., Liu Y. & Liu Z. P. Novel Chryseobacterium sp PYR2 degrades various organochlorine pesticides (OCPs) and achieves enhancing removal and complete degradation of DDT in highly contaminated soil. J. Environ. Manage. 161, 350–357 (2015). 26203874 10.1016/j.jenvman.2015.07.025

[b35] Bidlan R. & Manonmani H. K. Aerobic degradation of dichlorodiphenyltrichloroethane (DDT) by Serratia marcescens DT-1P. Process Biochem. 38, 49–56 (2002).

[b36] Santacruz G., Bandala E. R. & Torres L. G. Chlorinated pesticides (2,4-D and DDT) biodegradation at high concentrations using immobilized Pseudomonas fluorescens. J. Environ. Sci. Health., B. 40, 571–583 (2005). 16047880 10.1081/PFC-200061545

[b37] Kantachote D. . DDT resistance and transformation by different microbial strains isolated from DDT-contaminated soils and compost materials. Compost Sci. Util. 11, 300–310 (2003).

[b38] Sun G. D. . Biodegradation of dichlorodiphenyltrichloroethanes (DDTs) and hexachlorocyclohexanes (HCHs) with plant and nutrients and their effects on the microbial ecological kinetics. Microb. Ecol. 69, 281–292 (2015). 25213654 10.1007/s00248-014-0489-z

[b39] Chen M. . Anaerobic transformation of DDT related to iron (III) reduction and microbial community structure in paddy soils. J. Agricul. Food Chem. 61, 2224–2233 (2013). 10.1021/jf305029p 23402620

[b40] Bao P. . Dechlorination of p,p’-DDTs coupled with sulfate reduction by novel sulfate-reducing bacterium Clostridium sp BXM. Environ. Pollut. 162, 303–310 (2012). 22243878 10.1016/j.envpol.2011.11.037

[b41] Ba Y. . Ferrous ions accelerate sulfide-induced abiotic dechlorination of DDT in waterlogged paddy soil and in soil solution. J. Soils Sediments 11(7), 1209–1220 (2011).

[b42] Abed R. M. M., Al-Kindi S. & Al-Kharusi S. Diversity of bacterial communities along a petroleum contamination gradient in desert soils. Microb. Ecol. 69, 95–105 (2015). 25103912 10.1007/s00248-014-0475-5

[b43] Alexandrino M., Costa R., Canario A. V. M. & Costa M. C. Clostridia initiate heavy metal bioremoval in mixed sulfidogenic cultures. Environ. Sci. Technol. 48, 3378–3385 (2014). 24568215 10.1021/es4052044

[b44] Guo J. G. . Effect of historical residual hexachlorocyclohexanes and dichlorodiphenyltrichloroethane on bacterial communities in sediment core collected from an estuary in northeastern China by next-generation sequencing. Mar. Pollut. Bull. 93, 68–74 (2015). 25736815 10.1016/j.marpolbul.2015.02.013

[b45] Koblizkova M. . Can physicochemical and microbial soil properties explain enantiomeric shifts of chiral organochlorines? Environ. Sci. Technol. 42, 5978–5984 (2008). 18767654 10.1021/es800625d

[b46] Mueller T. A. & Kohler H. P. E. Chirality of pollutants-effects on metabolism and fate. Appl. Microbiol. Biotechnol. 64, 300–316 (2004). 14716466 10.1007/s00253-003-1511-4

