# Supplementary material for: Enantiomer signature and carbon isotope evidence for the migration and transformation of DDTs in arable soils across China
Source: Sci Rep. 2016 Dec 6;6:38475. doi: 10.1038/srep38475 (PMC5138824; doi:10.1038/srep38475)
Supplement: Supplementary Information [file srep38475-s1.doc]

**SUPPLEMENTORY INFORMATION**

Enantiomer signature and carbon isotope evidence for the migration and transformation of DDTs in arable soils across China

Lili Niu^1^, Chao Xu^2^, Siyu Zhu^1^, Huiming Bao^3^, Yang Xu^1^, Hongyi Li^1^, Zhijian Zhang^1^, Xichang Zhang^1^, Jiguo Qiu^1,2^, Weiping Liu^1,*^

^1^ International Joint Research Center for Persistent Toxic Substances (IJRC-PTS), MOE Key Laboratory of Environmental Remediation and Ecosystem Health, College of Environmental and Resource Sciences, Zhejiang University, Hangzhou 310058, China

^2^College of Environment, Zhejiang University of Technology, Hangzhou 310032, China

^3^Department of Geology & Geophysics, Louisiana State University, Baton Rouge, LA,

70803-4101, U.S.A.

Number of Pages: 28

Number of Text: 1

Number of Figures: 5

Number of Tables: 4

^*^Corresponding author: Weiping Liu

Tel./Fax: +86-0571-88982341. E-mail addresses: [wliu@zju.edu.cn](mailto:wliu@zju.edu.cn)

**TEXT S1 Materials and Methods**

**Sample Collection**

A total of 123 surface soils (0-20 cm) were collected, according to the distribution of cultivated land^1^, from 31 provinces, municipalities or autonomous across Mainland China in April and May 2013. The sites were marked by GPS and presented in Supplementary Figure 4. At each sites, overlying vegetation was excluded before surface soil samples were collected by a pre-clean stainless steel scoop. Five sub-samples were mixed to form a composite sample in an aluminum foil bag. After arriving at the laboratory in Zhejiang University, the soils were stored at -20°C. The soils were freeze-died, ground and sieved through 100 meshes (0.154 mm) before pretreatment.

**Sample Extraction and Analysis**

The analytical standards of 6 DDT compounds (*o,p’*-DDE, *p,p’*-DDE, *o,p’*-DDD, *p,p’*-DDD, *o,p’*-DDT and *p,p’*-DDT) purchased from Dr. Ehrenstorfer GmbH (Augsburg, Germany) and the mixture of surrogates (TCmX and PCB209) were from AccuStandard, Inc. (New Haven, CT, US). Other solvents and reagents of residue analysis grade were obtained from J&K Chemical Ltd. (Beijing, China). Anhydrous granular sodium sulfate, Florisil, silica gel, aluminum and super-purified copper powder were activated before use.

The procedures of sample pretreatment followed those in our previous study^2^. Briefly, surrogates and activated copper granules were firstly added into the samples. After Soxhlet-extracted with dichloromethane (DCM), the extract was solvent-exchanged into hexane, concentrated and cleaned up through a lab-made glass column. The column, with an inner diameter of 2 cm and a length of 40 cm, was packed with 2 g of anhydrous granular sodium sulfate (Na_2_SO4), 3.5 g of neutral silica gel, 4 g of alumina, 1.5 g of florisil and 2 g of Na_2_SO_4_ from bottom to top. Then, the target analytes eluted by hexane/DCM (7:3) were further concentrated to 0.5 mL. A known amount of pentachloronitrobenzene (PCNB) was used as the internal standard before injection.

The concentrations of DDT and its metabolites were measured on an Agilent 7890A gas chromatograph (GC-ECD, Agilent Technologies, Avondale, PA, USA). Colum of HP-5 (30m×0.25mm×0.25μm, Agilent Technologies Inc.) was used to for the separation of DDTs. The operation conditions were as follows: initial temperature at 80 °C, held for1 min, ramped to 200 °C at 10 °C min^-1^, then 1 °C min^-1^ to 225 °C, held for 1 min, finally climbed up to 260 °C at 15 °C min^-1^, held for 5 min; injector temperature, 250 °C; detector temperature, 300 °C. Nitrogen was used as the carrier gas with a flow rate of 1.0 mL min^-1^. The target compounds were recognized according to their retention times (RT) that were in the range of the mean RT ± 3 standard deviation (SD) for standards. The concentrations of several samples randomly selected were confirmed using a HP-5MS column (30 m × 0.32 mm × 0.25 μm, Agilent Technologies Inc., Santa Clara, CA) on a gas chromatograph-mass spectrometry (Agilent 7890A GC-5975C MS). The transfer line and ion source temperature were at 280 °C and 230 °C, respectively. The quantities of DDTs in samples were calculated using a seven-point calibration curve.

The enantiomer analysis of chiral *o,p’*-DDT was carried out using a GC-MS equipped with BGB-172 chiral capillary column (20% *tert*-butyldimenthylsilylated-*β*-cyclodextrin in OV-1701, 30m×0.25mm×0.25μm; BGB Analytik AG, Switzerland). The conditions were: initial oven-temperature of 90 °C for 1 min, climbed at 20 °C min^-1^ to 160 °C, 1 °C min^-1^ to 190 °C, held for 40 min, 25 °C min^-1^ to 225 °C, held for 40min and 250 °C and 280 °C for injector and detector temperatures, respectively. The carrier gas He was at a flow rate of 1.0 mL min^-1^. The transfer line and ion source temperature were at 280 °C and 230 °C, respectively. According to Yuan et al., the elution order of *o,p’*-DDT on the chiral BGB-172 column was (−) followed by (+)-enantiomer^3^.

To achieve enough amounts of targets for stable isotope analysis, a larger quantity of soils was used. The δ^13^C of DDT and its metabolites was measured using an Agilent 7890A GC coupled to a GV Isoprime IRMS (GV Instruments, UK) (GC-C-IRMS) via a modified GC5 combustion interface at College of Environmental and Resource Sciences, Zhejiang University. The column and instrumental conditions were the same as those for DDT analysis by GC-MS. The δ^13^C values of target compounds were calculated with the following equation.

$\delta{}^{13}C=\left(\frac{R_{sample}}{R_{s\tan dard}}-1\right)\times1000$‰ (1)

where R_sample_ and R_standard_ are the carbon isotope ratio (^13^C/^12^C) for the sample and the standard (V-PDB), respectively.

Due to the limitation of concentration and the existence of interfering substances, only the δ^13^C of *p,p’*-DDE and *o,p’*-DDT were determined.

**DNA extraction and Illumina Miseq sequencing**

Soil DNA was isolated from 0.25 g fresh soil samples using Power Soil DNA kit (Mo Bio Laboratories, USA) based on the instructions of the manufacturer. The extracted DNA was checked by electrophoresis using a 1.2% agarose gel. Total DNA was used for sequencing of 16S ribosomal RNA (rRNA) in Shenzhen Huada Genomics Institute using Illumina Miseq (300 bp paired-end). The V3 and V4 hypervariable regions of the bacterial 16S rRNA gene were selected to sequence. All reads obtained from DNA samples after sequencing were selected and assembled to tags. High quality tags were selected for statistical analysis. Operational taxonomic units were clustered using a sequence similarity of 97%. The comparison of sequences was conducted by means of Basic Local Alignment Search Tool on the NCBI’s non-redundant (nr) database.The sequencing data were all deposited in GenBank (Accession Number: SRP072159).

**Statistical treatment**

The nationwide distributions of DDT and its metabolites were mapped using geographic information system (GIS) software (ArcGIS 9.3, ESRI, Redlands, California). The relationships between DDT and microbial communities were explored by Network analysis. Package CREPE in R was used to calculate the compositionality-corrected p-values and q-values for the bacterial and DDTs composition data, while Spearman correlation was used as similarity measure ^4^. Higher-rank taxa were filtered if one of their members occupied 99% of their abundances. Only pairs with a significant *p*-value lower than 0.01 were visualized as a network diagram using Cytoscape 3.3.

Table S1. Enantiomeric Fractions (EFs) of *o,p’*-DDT in Soils from Four Regions of Mainland China.

|  | n | Mean | Percentage (%) | |
| --- | --- | --- | --- | --- |
|  |  |  | EF > 0.5 | EF < 0.5 |
| East China | 34 | 0.553 | 55.9 | 44.1 |
| Central China | 23 | 0.445 | 34.8 | 65.2 |
| West China | 28 | 0.484 | 42.9 | 57.1 |
| Northeast China | 9 | 0.648 | 77.8 | 22.2 |

Table S2. The δ^13^C Values of *p,p’*-DDE and *o,p’*-DDT and Environmental Parameters of Selected Soil Samples

| City | Location | Latitude (°N) | Longitude (°E) | Elevation (m) | Temperature (°C) | C*p,p’*-DDE-OM (ng/g) | C*p,p’*-DDT (ng/g) | C*o,p’*-DDT (ng/g) | EF of *o,p’*-DDT | δ^13^C±SD (‰*,* *p,p*’-DDE) | δ^13^C±SD (‰*,* *o,p*’-DDT) |
| --- | --- | --- | --- | --- | --- | --- | --- | --- | --- | --- | --- |
| Shanghai | East China | 30.98 | 121.1 | 10 | 16.9 | 1.52 | 15.9 | 0.223 | 0.420 | -30.26±0.06 | -31.46±0.14 |
| Shangluo, Shannxi | Northwest China | 33.67 | 109.11 | 775 | 13.5 | 0.995 | 1.78 | 0.184 | 0.635 | -28.14±0.32 | -31.92±0.06 |
| Wulumuqi, Xinjiang | Northwest China | 43.96 | 87.64 | 604 | 7.4 | 0.148 | 2.01 | 0.464 | 0.710 | -25.45±0.04 | -31.60±0.28 |
| Linzhi, Tibet | Northwest China | 29.64 | 94.35 | 3000 | 8.7 | 0.0346 | 0.041 | 0.021 | 0.488 | -24.81±0.12 | -25.89±0.06 |
| Tacheng, Xinjiang | Northwest China | 46.34 | 86.24 | 596 | 6.0 | 1.17 | 0.870 | 0.327 | 0.618 | -26.49±0.26 | -32.40±0.07 |
| Yulin, Guangxi | South China | 22.72 | 110.13 | 83 | 21.0 | 1.55 | 0.626 | 0.439 | 0.416 | -28.37±0.16 | -27.93±0.36 |
| Guangan, Sichuan | Central China | 30.04 | 107.02 | 308 | 16.0 | 1.71 | 0.277 | 0.020 | 0.477 | -27.0±0.11 | -30.17±0.09 |
| Wenzhou, Zhejiang | East China | 27.78 | 120.6 | 13 | 18.3 | 1.63 | 0.255 | 0.027 | 0.848 | -27.78±0.25 | -31.77±0.09 |
| Dalian, Liaoning | Northeast China | 39.6 | 122.24 | 54 | 10.5 | 0.218 | 0.383 | 1.11 | 0.567 | -27.46±0.12 | -32.57±0.28 |
| Nanning, Guangxi | South China | 22.77 | 108.21 | 46 | 21.4 | 0.017 | 0.016 | 0.042 | 0.333 | -28.32±0.34 | -27.02±0.08 |
| Standard Product | - | - | - | - | - | - |  | - |  | -28.77±0.06 | -34.23±0.13 |

Table S3. Bacterial Taxa and Corresponding Stereisomers of DDT and Its Metabolites in Soils.

| No. | positive correlate | negative correlate | size | Taxon |
| --- | --- | --- | --- | --- |
| 1053 | (-)-*o,p'*-DDT |  | 0.0001 | Bacteria; Actinobacteria; Actinobacteria; Actinomycetales; Nocardiaceae; Nocardia; Nocardia_sienata |
| 1536 |  |  | 0.0044 | Bacteria; Proteobacteria; Gammaproteobacteria; Pseudomonadales; Pseudomonadaceae; Cellvibrio; Cellvibrio_fibrivorans |
| 69 |  |  | 0.4347 | Bacteria; Proteobacteria; Alphaproteobacteria |
| 995 | (+)-*o,p'*-DDT | *p,p'*-DDD | 0.0007 | Bacteria; Actinobacteria; Actinobacteria; Acidimicrobiales; Iamiaceae; Iamia; Iamia_majanohamensis |
| 166 |  |  | 0.0029 | Bacteria; Proteobacteria; Gammaproteobacteria; Alteromonadales |
| 996 |  |  | 0.0127 | Bacteria; Actinobacteria; Actinobacteria; Acidimicrobiales; Other; Other; Other |
| 1346 |  |  | 0.0151 | Bacteria; Proteobacteria; Alphaproteobacteria; Sphingomonadales; Erythrobacteraceae; Other; Other |
| 1301 | *o,p'*-DDD |  | 0.0002 | Bacteria; Proteobacteria; Alphaproteobacteria; Rhizobiales; Rhizobiaceae; Kaistia; Kaistia_terrae |
| 1382 |  |  | 0.0008 | Bacteria; Proteobacteria; Betaproteobacteria; Burkholderiales; Comamonadaceae; Hydrogenophaga; Hydrogenophaga_caeni |
| 1736 |  |  | 0.0020 | Bacteria; Firmicutes; Negativicutes; Selenomonadales; Veillonellaceae; Veillonellaceae_genus_incertae_sedis; Psychrosinus_fermentans |
| 324 |  |  | 0.0021 | Bacteria; Proteobacteria; Betaproteobacteria; Burkholderiales; Burkholderiales_incertae_sedis |
| 956 |  |  | 0.0083 | Bacteria; Firmicutes; Clostridia; Clostridiales; Ruminococcaceae; Other |
| 296 | *o,p'*-DDE | *o,p'*-DDT, (+)-*o,p'*-DDT | 0.0203 | Bacteria; Nitrospira; Nitrospira; Nitrospirales; Nitrospiraceae |
| 167 |  |  | 0.0014 | Bacteria; Proteobacteria; Gammaproteobacteria; Chromatiales |
| 1099 | *o,p'*-DDT | *p,p'*-DDE | 0.0002 | Bacteria; Actinobacteria; Actinobacteria; Actinomycetales; Sporichthyaceae; Sporichthya; Sporichthya_polymorpha |
| 1185 |  |  | 0.0005 | Bacteria; Bacteroidetes; Sphingobacteria; Sphingobacteriales; Cytophagaceae; Other; Other |
| 1191 |  |  | 0.0015 | Bacteria; Bacteroidetes; Sphingobacteria; Sphingobacteriales; Cytophagaceae; Rhodocytophaga; Rhodocytophaga_aerolata |
| 1172 |  |  | 0.0021 | Bacteria; Bacteroidetes; Sphingobacteria; Sphingobacteriales; Cytophagaceae; Adhaeribacter; Adhaeribacter_aquaticus |
| 1325 |  |  | 0.0023 | Bacteria; Proteobacteria; Alphaproteobacteria; Rhodospirillales; Acetobacteraceae; Roseomonas; Roseomonas_aquatica |
| 334 |  |  | 0.0024 | Bacteria; Proteobacteria; Deltaproteobacteria; Bdellovibrionales; Bacteriovoracaceae |
| 683 |  |  | 0.0038 | Bacteria; Proteobacteria; Alphaproteobacteria; Rhodospirillales; Acetobacteraceae; Roseomonas |
| 1319 |  |  | 0.0050 | Bacteria; Proteobacteria; Alphaproteobacteria; Rhodobacterales; Rhodobacteraceae; Rubellimicrobium; Rubellimicrobium_aerolatum |
| 679 |  |  | 0.0053 | Bacteria; Proteobacteria; Alphaproteobacteria; Rhodobacterales; Rhodobacteraceae; Rubellimicrobium |
| 1556 |  |  | 0.0055 | Bacteria; Proteobacteria; Gammaproteobacteria; Xanthomonadales; Xanthomonadaceae; Arenimonas; Other |
| 299 |  |  | 0.0140 | Bacteria; Proteobacteria; Alphaproteobacteria; Caulobacterales; Caulobacteraceae |
| 277 |  |  | 0.0179 | Bacteria; Bacteroidetes; Sphingobacteria; Sphingobacteriales; Cytophagaceae |
| 630 |  |  | 0.0225 | Bacteria; Gemmatimonadetes; Gemmatimonadetes; Gemmatimonadales; Gemmatimonadaceae; Gemmatimonas |
| 122 |  |  | 0.1209 | Bacteria; Bacteroidetes; Sphingobacteria; Sphingobacteriales |
| 12 |  |  | 0.1819 | Bacteria; Bacteroidetes |
| 846 | *o,p'*-DDT, (-)-*o,p'*-DDT |  | 0.006142 | Bacteria; Proteobacteria; Gammaproteobacteria; Pseudomonadales; Pseudomonadaceae; Cellvibrio |
| 1313 |  |  | 0.007881 | Bacteria; Proteobacteria; Alphaproteobacteria; Rhodobacterales; Rhodobacteraceae; Other; Other |
| 1340 |  |  | 0.034614 | Bacteria; Proteobacteria; Alphaproteobacteria; Rhodospirillales; Rhodospirillaceae; Skermanella; Skermanella_aerolata |
| 317 |  |  | 0.048793 | Bacteria; Proteobacteria; Alphaproteobacteria; Rhodospirillales; Rhodospirillaceae |
| 1280 |  |  | 0.002768 | Bacteria; Proteobacteria; Alphaproteobacteria; Rhizobiales; Hyphomicrobiaceae; Devosia; Other |
| 1264 |  |  | 0.002934 | Bacteria; Proteobacteria; Alphaproteobacteria; Caulobacterales; Caulobacteraceae; Brevundimonas; Brevundimonas_alba |
| 639 |  |  | 0.003298 | Bacteria; Proteobacteria; Alphaproteobacteria; Caulobacterales; Caulobacteraceae; Brevundimonas |
| 1004 | *o,p'*-DDT, (+)-*o,p'*-DDT |  | 0.000776 | Bacteria; Actinobacteria; Actinobacteria; Actinomycetales; Cellulomonadaceae; Other; Other |
| 1174 |  |  | 0.002109 | Bacteria; Bacteroidetes; Sphingobacteria; Sphingobacteriales; Cytophagaceae; Adhaeribacter; Other |
| 650 |  |  | 0.013642 | Bacteria; Proteobacteria; Alphaproteobacteria; Rhizobiales; Hyphomicrobiaceae; Devosia |
| 1427 | *o,p'*-DDT, (+)-*o,p'*-DDT, (-)-*o,p'*-DDT |  | 0.002126 | Bacteria; Proteobacteria; Deltaproteobacteria; Bdellovibrionales; Bacteriovoracaceae; Peredibacter; Peredibacter_starrii |
| 314 |  |  | 0.015423 | Bacteria; Proteobacteria; Alphaproteobacteria; Rhodobacterales; Rhodobacteraceae |
| 972 | *p,p'*-DDD |  | 0.0002 | Bacteria; Acidobacteria; Acidobacteria_Gp13; Acidobacteria_Gp13_order_incertae_sedis; Acidobacteria_Gp13_family_incertae_sedis;  Gp13; Other |
| 1377 |  |  | 0.0008 | Bacteria; Proteobacteria; Betaproteobacteria; Burkholderiales; Burkholderiales_incertae_sedis; Other; Other |
| 597 |  |  | 0.0013 | Bacteria; Bacteroidetes; Sphingobacteria; Sphingobacteriales; Sphingobacteriaceae; Mucilaginibacter |
| 1704 |  |  | 0.0022 | Bacteria; Firmicutes; Clostridia; Clostridiales; Lachnospiraceae; Clostridium_XlVa; Other |
| 1579 |  |  | 0.0048 | Bacteria; Proteobacteria; Gammaproteobacteria; Xanthomonadales; Xanthomonadaceae; Stenotrophomonas; Stenotrophomonas_rhizophila |
| 867 |  |  | 0.0055 | Bacteria; Proteobacteria; Gammaproteobacteria; Xanthomonadales; Xanthomonadaceae; Stenotrophomonas |
| 713 |  |  | 0.0055 | Bacteria; Proteobacteria; Betaproteobacteria; Burkholderiales; Burkholderiaceae; Burkholderia |
| 323 |  |  | 0.0077 | Bacteria; Proteobacteria; Betaproteobacteria; Burkholderiales; Burkholderiaceae |
| 967 |  |  | 0.0103 | Bacteria; Acidobacteria; Acidobacteria_Gp1; Acidobacteria_Gp1_order_incertae_sedis; Acidobacteria_Gp1_family_incertae_sedis; Gp1;  Other |
| 431 |  |  | 0.0105 | Bacteria; Acidobacteria; Acidobacteria_Gp1; Acidobacteria_Gp1_order_incertae_sedis; Acidobacteria_Gp1_family_incertae_sedis; Gp1 |
| 1488 |  |  | 0.0124 | Bacteria; Proteobacteria; Gammaproteobacteria; Enterobacteriales; Enterobacteriaceae; Other; Other |
| 1726 |  |  | 0.0159 | Bacteria; Firmicutes; Clostridia; Clostridiales; Ruminococcaceae; Clostridium_III; Other |
| 954 |  |  | 0.0165 | Bacteria; Firmicutes; Clostridia; Clostridiales; Ruminococcaceae; Clostridium_III |
| 422 |  |  | 0.0276 | Bacteria; Firmicutes; Clostridia; Clostridiales; Ruminococcaceae |
| 361 |  |  | 0.0135 | Bacteria; Proteobacteria; Gammaproteobacteria; Enterobacteriales; Enterobacteriaceae |
| 1473 | *p,p'*-DDE | (+)-*o,p'*-DDT | 0.0795 | Bacteria; Proteobacteria; Deltaproteobacteria; Other; Other; Other; Other |
| 1115 |  | *o,p'*-DDT,*p,p'*-DDT | 0.0003 | Bacteria; Actinobacteria; Actinobacteria; Coriobacteriales; Coriobacteriaceae; Other; Other |
| 257 |  |  | 0.0008 | Bacteria; Actinobacteria; Actinobacteria; Coriobacteriales; Coriobacteriaceae |
| 804 |  |  | 0.0020 | Bacteria; Proteobacteria; Deltaproteobacteria; Syntrophobacterales; Syntrophobacteraceae; Syntrophobacter |
| 15 |  |  | 0.0027 | Bacteria; Chloroflexi |
| 160 |  |  | 0.0046 | Bacteria; Proteobacteria; Deltaproteobacteria; Syntrophobacterales |
| 1250 |  | *o,p'*-DDT,*p,p'*-DDT,(+)*-o,p'*-DDT | 0.0026 | Bacteria; Chloroflexi; Other; Other; Other; Other; Other |
| 1482 |  | *p,p'*-DDT | 0.0013 | Bacteria; Proteobacteria; Deltaproteobacteria; Syntrophobacterales; Syntrophobacteraceae; Syntrophobacter; Other |
| 357 |  |  | 0.0013 | Bacteria; Proteobacteria; Deltaproteobacteria; Syntrophobacterales; Syntrophaceae |
| 1258 |  |  | 0.0068 | Bacteria; Nitrospira; Nitrospira; Nitrospirales; Nitrospiraceae; Thermodesulfovibrio; Other |
| 1447 |  |  | 0.0009 | Bacteria; Proteobacteria; Deltaproteobacteria; Desulfuromonadales; Geobacteraceae; Geobacter; Geobacter_argillaceus |
| 358 |  |  | 0.0031 | Bacteria; Proteobacteria; Deltaproteobacteria; Syntrophobacterales; Syntrophobacteraceae |
| 1452 |  |  | 0.0037 | Bacteria; Proteobacteria; Deltaproteobacteria; Desulfuromonadales; Geobacteraceae; Geobacter; Other |
| 776 |  |  | 0.0057 | Bacteria; Proteobacteria; Deltaproteobacteria; Desulfuromonadales; Geobacteraceae; Geobacter |
| 157 |  |  | 0.0064 | Bacteria; Proteobacteria; Deltaproteobacteria; Desulfuromonadales |
| 1512 |  |  | 0.0072 | Bacteria; Proteobacteria; Gammaproteobacteria; Legionellales; Coxiellaceae; Aquicella; Aquicella_siphonis |
| 1581 |  |  | 0.0558 | Bacteria; Proteobacteria; Other; Other; Other; Other; Other |
| 71 |  |  | 0.2452 | Bacteria; Proteobacteria; Deltaproteobacteria |
| 994 | *p,p'*-DDT | *p,p'*-DDD | 0.0053 | Bacteria; Actinobacteria; Actinobacteria; Acidimicrobiales; Acidimicrobiaceae; Ilumatobacter; Ilumatobacter_fluminis |
| 1128 |  |  | 0.0090 | Bacteria; Bacteroidetes; Bacteroidetes_incertae_sedis_class_incertae_sedis; Bacteroidetes_incertae_sedis_order_incertae_sedis; Bacteroidetes_incertae_sedis_family_incertae_sedis; Ohtaekwangia; Ohtaekwangia_koreensis |
| 110 | *p,p'*-DDT*,* (+)-*o,p'*-DDT |  | 0.019010 | Bacteria; Actinobacteria; Actinobacteria; Acidimicrobiales |
| 156 |  | *o,p'*-DDT | 0.0003 | Bacteria; Proteobacteria; Deltaproteobacteria; Desulfovibrionales |
| 1484 |  |  | 0.0006 | Bacteria; Proteobacteria; Deltaproteobacteria; Syntrophobacterales; Syntrophobacteraceae; Syntrophobacter; Syntrophobacter_wolinii |
| 1675 |  |  | 0.0020 | Bacteria; Firmicutes; Clostridia; Clostridiales; Clostridiaceae_1; Clostridium_sensu_stricto; Clostridium_aurantibutyricum |
| 1729 |  |  | 0.0083 | Bacteria; Firmicutes; Clostridia; Clostridiales; Ruminococcaceae; Other; Other |
| 1685 |  |  | 0.0335 | Bacteria; Firmicutes; Clostridia; Clostridiales; Clostridiaceae_1; Clostridium_sensu_stricto; Other |
| 184 |  |  | 0.1439 | Bacteria; Firmicutes; Clostridia; Clostridiales |
| 1740 |  |  | 0.1539 | Bacteria; WS3; WS3_class_incertae_sedis; WS3_order_incertae_sedis; WS3_family_incertae_sedis; WS3_genus_incertae_sedis; Other |
| 86 |  |  | 0.1585 | Bacteria; WS3; WS3_class_incertae_sedis |
| 27 |  |  | 0.1682 | Bacteria; WS3 |
| 1547 |  | *o,p'*-DDT, (-)-*o,p'*-DDT | 0.0011 | Bacteria; Proteobacteria; Gammaproteobacteria; Thiotrichales; Thiotrichales_incertae_sedis; Caedibacter; Caedibacter_caryophilus |
| 983 |  | *o,p'*-DDT,(+)-*o,p'*-DDT | 0.0133 | Bacteria; Acidobacteria; Acidobacteria_Gp3; Acidobacteria_Gp3_order_incertae_sedis; Acidobacteria_Gp3_family_incertae_sedis; Gp3;  Other |
| 1679 |  | *o,p'*-DDT,*p,p'*-DDT | 0.0060 | Bacteria; Firmicutes; Clostridia; Clostridiales; Clostridiaceae_1; Clostridium_sensu_stricto; Clostridium_magnum |
| 924 |  |  | 0.0570 | Bacteria; Firmicutes; Clostridia; Clostridiales; Clostridiaceae_1; Clostridium_sensu_stricto |
| 411 |  |  | 0.0632 | Bacteria; Firmicutes; Clostridia; Clostridiales; Clostridiaceae_1 |
| 1103 |  | *p,p'*-DDD | 0.0003 | Bacteria; Actinobacteria; Actinobacteria; Actinomycetales; Streptosporangiaceae; Microbispora; Microbispora_corallina |
| 1466 |  |  | 0.0035 | Bacteria; Proteobacteria; Deltaproteobacteria; Myxococcales; Nannocystaceae; Other; Other |
| 969 |  |  | 0.0066 | Bacteria; Acidobacteria; Acidobacteria_Gp10; Acidobacteria_Gp10_order_incertae_sedis; Acidobacteria_Gp10_family_incertae_sedis;  Gp10; Other |
| 988 |  |  | 0.0067 | Bacteria; Acidobacteria; Acidobacteria_Gp7; Acidobacteria_Gp7_order_incertae_sedis; Acidobacteria_Gp7_family_incertae_sedis; Gp7;  Other |
| 351 |  |  | 0.0086 | Bacteria; Proteobacteria; Deltaproteobacteria; Myxococcales; Nannocystaceae |
| 1294 |  |  | 0.0145 | Bacteria; Proteobacteria; Alphaproteobacteria; Rhizobiales; Methylobacteriaceae; Microvirga; Other |
| 1456 |  |  | 0.0147 | Bacteria; Proteobacteria; Deltaproteobacteria; Myxococcales; Cystobacteraceae; Archangium; Archangium_gephyra |
| 555 |  |  | 0.0161 | Bacteria; Bacteroidetes; Bacteroidetes_incertae_sedis_class_incertae_sedis; Bacteroidetes_incertae_sedis_order_incertae_sedis;  Bacteroidetes_incertae_sedis_family_incertae_sedis; Ohtaekwangia |
| 306 |  |  | 0.0189 | Bacteria; Proteobacteria; Alphaproteobacteria; Rhizobiales; Methylobacteriaceae |
| 1391 |  |  | 0.0199 | Bacteria; Proteobacteria; Betaproteobacteria; Burkholderiales; Other; Other; Other |
| 1553 |  |  | 0.0856 | Bacteria; Proteobacteria; Gammaproteobacteria; Xanthomonadales; Sinobacteraceae; Steroidobacter; Steroidobacter_denitrificans |
| 385 |  |  | 0.0876 | Bacteria; Proteobacteria; Gammaproteobacteria; Xanthomonadales; Sinobacteraceae |
| 1094 |  | *p,p'*-DDE | 0.0002 | Bacteria; Actinobacteria; Actinobacteria; Actinomycetales; Pseudonocardiaceae; Saccharothrix; Saccharothrix_texasensis |
| 1077 |  |  | 0.0064 | Bacteria; Actinobacteria; Actinobacteria; Actinomycetales; Nocardioidaceae; Other; Other |
| 177 |  | *p,p'*-DDT | 0.0035 | Bacteria; Spirochaetes; Spirochaetes; Spirochaetales |
| 379 |  |  | 0.0044 | Bacteria; Proteobacteria; Gammaproteobacteria; Pseudomonadales; Moraxellaceae |
| 1455 |  |  | 0.0064 | Bacteria; Proteobacteria; Deltaproteobacteria; Myxococcales; Cystobacteraceae; Anaeromyxobacter; Anaeromyxobacter_dehalogenans |

Table S4. Concentrations of DDT and Its Metabolites in Arable Soils from Other Studies in China and the World (ng/g, soil)

| Site | *o,p*’-DDE | *p,p*’-DDE | *o,p*’-DDD | *p,p*’-DDD | *o,p*’-DDT | *p,p*’-DDT | ∑DDTs | Sampling time | Reference |
| --- | --- | --- | --- | --- | --- | --- | --- | --- | --- |
| Yangtze River Delta, China | 2.5 | 6.2 | 0.9 | 1.3 | 8.0 | 56.1 | 59.3 | 2014 | 5 |
| Jilin, China | - | 2.95 | - | 3.17 | 2.31 | 4.76 | 13.19 | 2013 | 6 |
| Zhejiang, China | - | 21 | 10 | 20 | 21 | 9.6 | 82 | 2006 | 7 |
| Shanghai, China | - | 16.14 | - | 4.56 | 1.66 | 3.26 | 21.41 | 2007 | 8 |
| Zhangzhou, China | - | 2.38 | - | 0.65 | 2.91 | 3.86 | 3.86 | 2009 | 9 |
| Hongze Lake, China | - | 12.82 | - | 6.27 | 3.43 | 7.55 | 30.07 | 2009 | 10 |
| Ningde, China | - | - | - | - | - | - | 29.61 | 2009 | 11 |
| Guangzhou, China | - | 24.53 | - | 11.60 | - | 23.27 | 67.32 | 2004 | 12 |
| Tianjin, China | 1.31 | 22.5 | 5.00 | 4.57 | 3.01 | 1.84 | 38.23 | 2008 | 13 |
| Tianjin, China | 9.95 | 84 | 1.78 | 17.5 | 2.55 | 11.13 | 127 | 2002 | 14 |
| Beijing, China | - | 39.72 | - | 2.68 | 8.39 | 13.66 | 64.44 | 2001 | 15 |
| Beijing, China | - | - | - | - | - | - | 3.4-2910.0 | 2003 | 16 |
| Central Jilin, China | - | 1.80 | - | 0.44 | 0.39 | 0.38 | 3.01 | 2007 ^a^ | 17 |
| Anhui, China | 3.78 | - | - | 13.83 | - | - | - | 2010 | 18 |
| Hunan, China | - | 19.64 | - | 7.23 | 2.34 | 7.67 | 36.26 | 2009 | 19 |
| South of Jiangsu, China | - | 77.7 | - | 11.9 | 9.3 | 64.4 | 163.2 | 2004 ^a^ | 20 |
| North of Jiangsu, China | 0.765 | 6.13 | - | 1.285 | 2.275 | 3.245 | 13.71 | 2006 | 21 |
| Wuhan, China | 2.62 | 73.38 | 2.94 | 9.26 | 10.64 | 52.74 | 151.56 | 2009 | 22 |
| Nanjing, China | - | 32.4 | - | 4.6 | 6.9 | 20.2 | 64.1 | 2002，2003 | 23 |
| Pearl River Delta, China | - | 20.65 | - | 15.31 | 3.625 | 11.94 | - | 2002 | 24 |
| Rio Verde region  of San Luis Potosi, Mexico | - | 3.61 | - | 7.80 | - | 70.53 | - | 2014^a^ | 25 |
| Osogbo, Nigeria | - | 54.32 | 52.77 | 43.81 | - | 62.44 | - | 2004, 2005 | 26 |
| Dehradun, India | - | 0.073 | - | 0.0332 | 0.003 | 0.005 | 0.117 | 2003 ^a^ | 27 |
| Dibrugarh, India | 35 | 237 | - | 56 | 122 | 307 | 757 | 2009, 2010 | 28 |
| Nagaon, India | 51 | 276 | - | 73 | 150 | 351 | 903 | 2009, 2010 | 28 |
| Romania | - | - | - | - | - | - | 35.5 | 2001 ^a^ | 29 |
| Hanoi, Vietnam | - | 48.10 | - | 23.73 | - | 18.04 | 89.86 | 2006 | 30 |
| Bacninh, Vietnam | - | 52.84 | - | 29.04 | - | 24.93 | 106.79 | 2006 | 31 |
| Czech Republic | - | 48.46 | - | 6.85 | - | 58.36 | 113.7 | Since1992 | 32 |
| Puebla and Mexico, Mexican Republic | - | 5.3 | - | - | 16.1 | 52.2 | 70.5 | 2006 | 33 |
| Leipzig–Halle region, Germany | - | - | - | - | - | 39.43 | 72.00 | 1995, 1996 | 34 |
| Georgia, USA | - | 5.58 | - | 0.53 | - | 3.63 | 8.75 | 2003 | 35 |
| South Carolina, USA | - | 7 | - | 0.69 | - | 3.79 | 11.18 | 2003 | 35 |
| Southern United States | 1.9 | 143 | - | 2 | 13 | 60 | 211 | 1999-2000 | 36 |
| South-western Spain | 0.03 | 1.17 | 0.12 | 0.11 | 0.04 | 0.32 | 1.79 | 2007,2008 | 37 |
| British Columbia, Canada | 6.64 | 146.7 | 56.50 | 188.1 | 254.4 | 660.4 | 1312.4 | 1999， 2000 | 38 |
| Brazzaville, Congo | - | 40.225 | - | 2.523 | 10.8 | 125.5 | 179 | 1999 | 39 |
| Whole China | 0.137 | 3.29 | 0.242 | 0.469 | 0.711 | 3.21 | 8.06 | 2013 | This study |

^a^ The sampling time was replaced by publication date because of the lack of information.


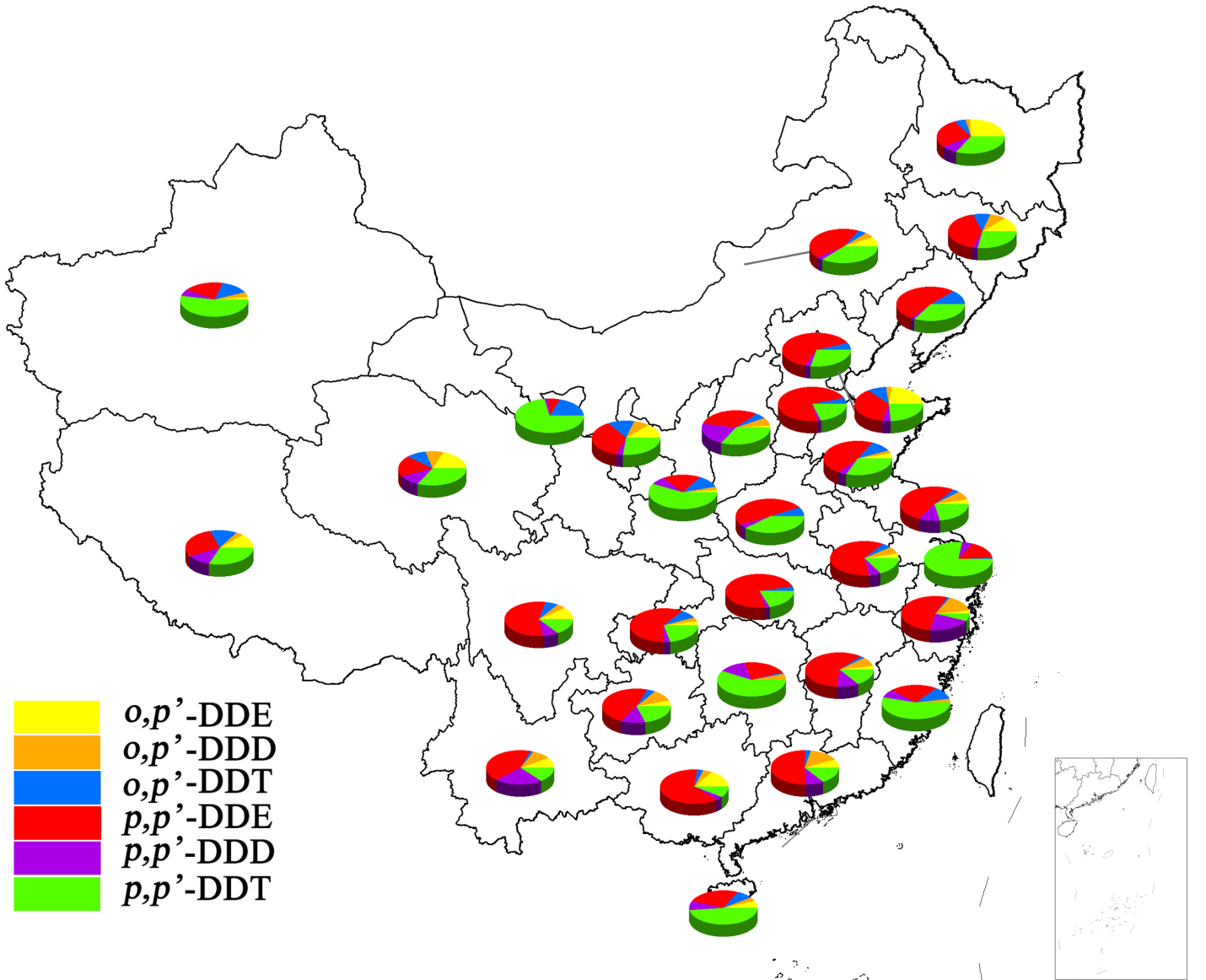


Figure S1. Residue profiles of DDT and its metabolites in soils across Mainland China. The map was created using ArcGIS 9.3 software (ESRI, Redlands, California, USA, http://www.esri.com/software/arcgis/arcgis-for-desktop). *Scientific Reports* remains neutral with regard to jurisdictional claims in published maps.


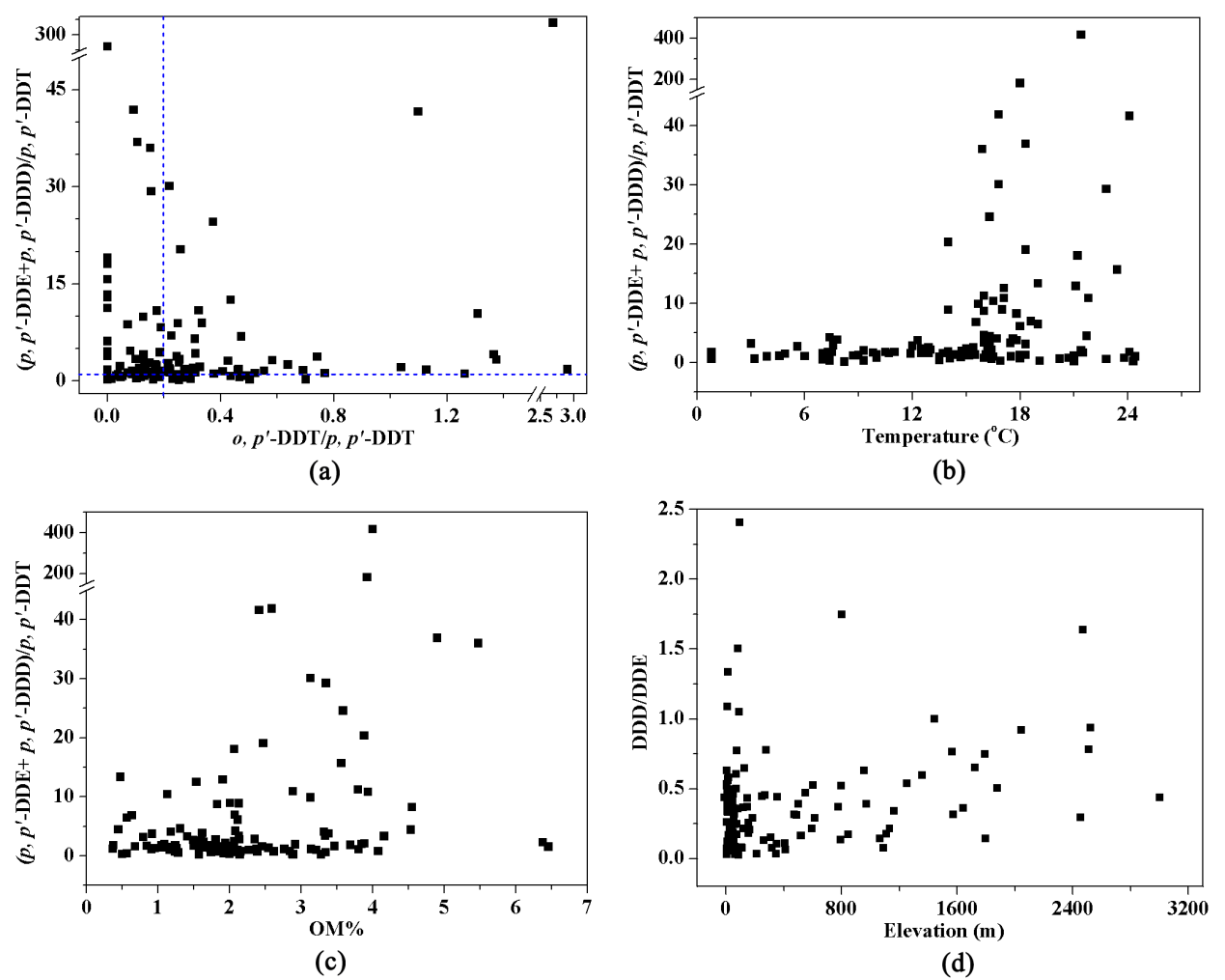


Figure S2. The ratios of DDT components in soils (a) and their relationships with temperature (b), soil organic matter (c) and elevation (d) of the sampling sites.


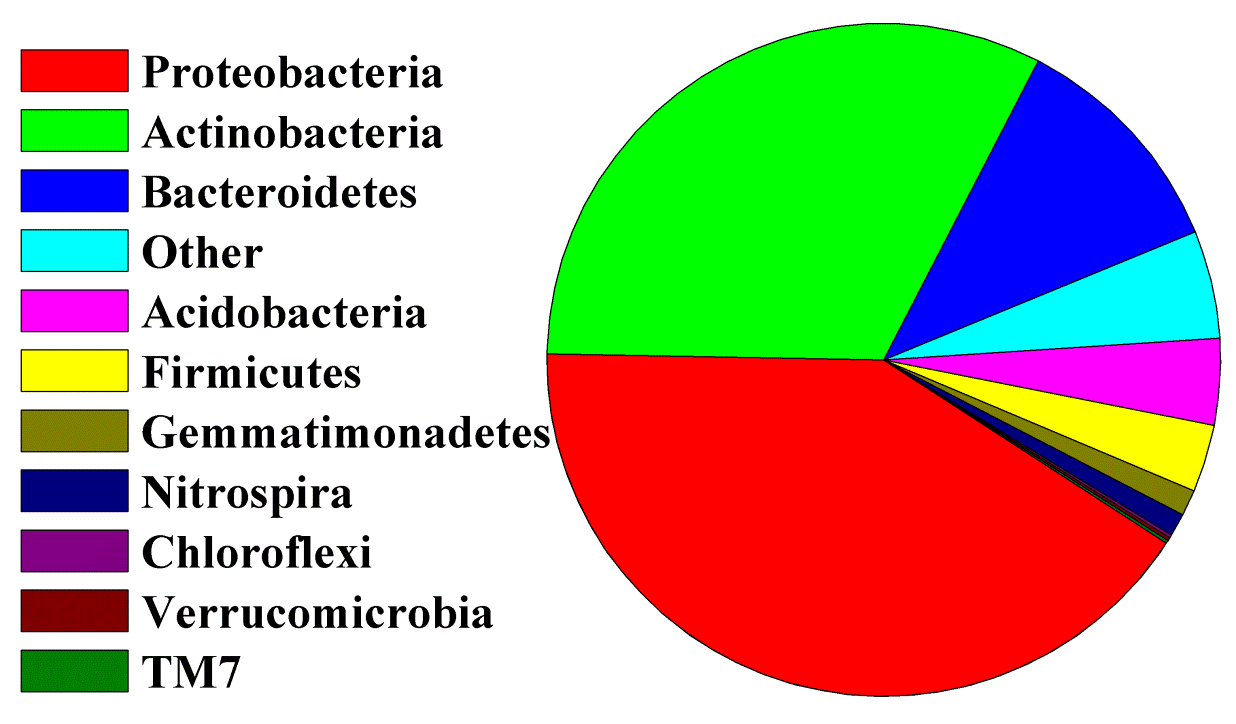


Figure S3. The composition of bacterial communities in arable soils across Mainland China at the level of phylum.


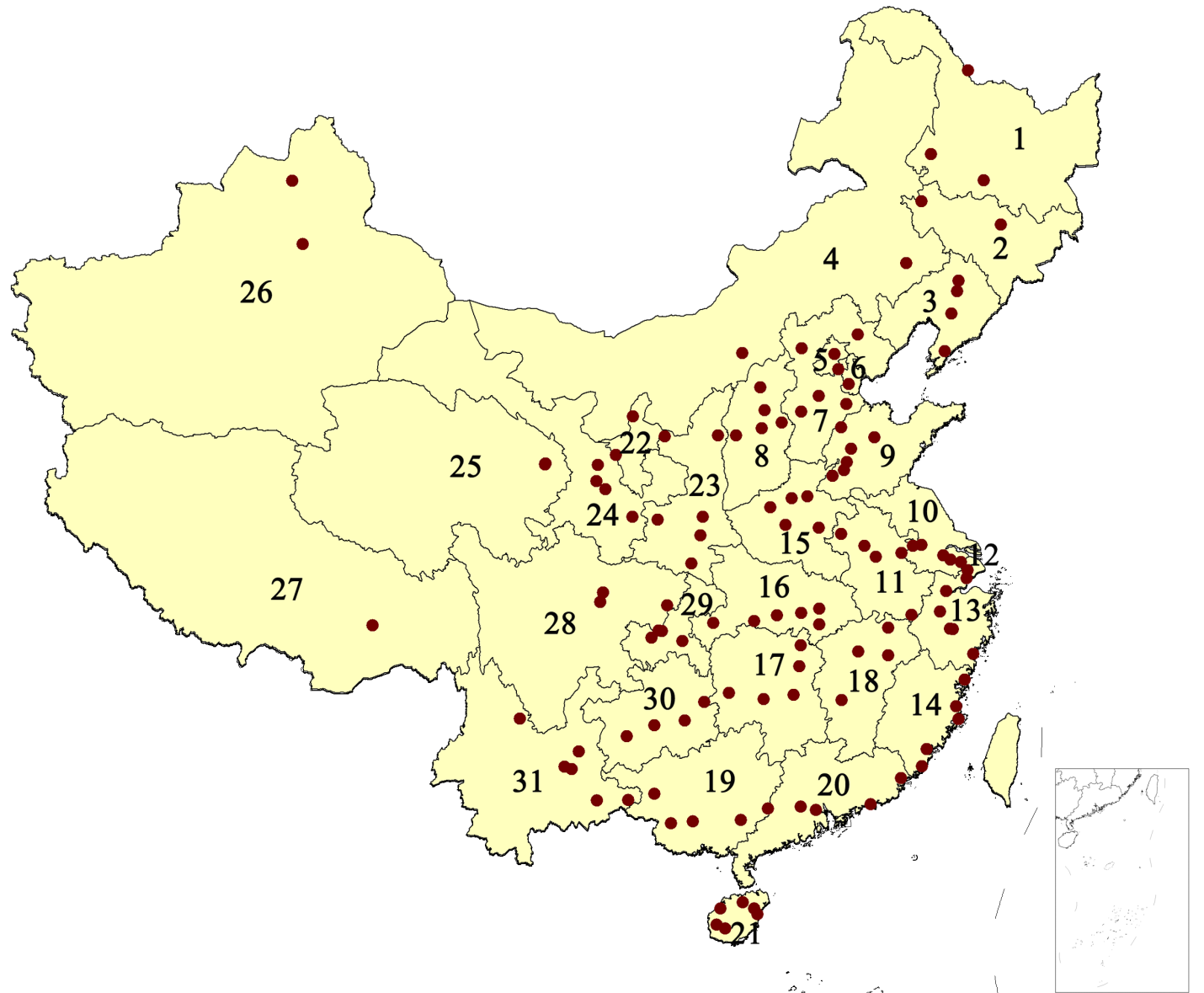


Figure S4. Distribution of soil sampling sites in arable soils in Mainland China (n=123). The number 1-31 represent (1) Heilongjiang, (2) Jilin, (3) Liaoning, (4) Inner Mongolia, (5) Beijing, (6) Tianjin, (7) Hebei, (8) Shanxi, (9) Shandong, (10) Jiangsu, (11) Anhui, (12) Shanghai, (13) Zhejiang, (14) Fujian, (15) Henan, (16) Hubei, (17) Hunan, (18) Jiangxi, (19) Guangxi, (20) Guangdong, (21) Hainan, (22) Ningxia, (23) Shaanxi, (24) Gansu, (25) Qinghai, (26) Xinjiang, (27) Tibet, (28) Sichuan, (29) Chongqing, (30) Guizhou, and (31) Yunnan Province, respectively. The map was created using ArcGIS 9.3 software (ESRI, Redlands, California, USA, http://www.esri.com/software/arcgis/arcgis-for-desktop). *Scientific Reports* remains neutral with regard to jurisdictional claims in published maps.

| 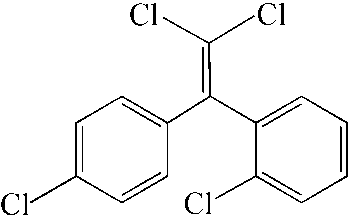 | 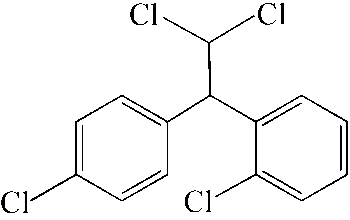 | 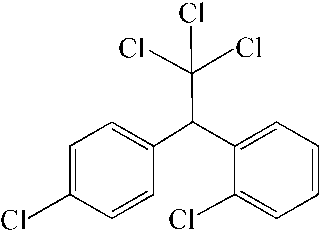 |
| --- | --- | --- |
| *o, p’*-DDE | *o, p’*-DDD | *o, p’*-DDT |
| 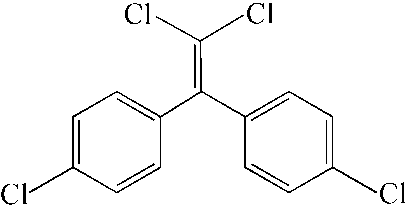 | 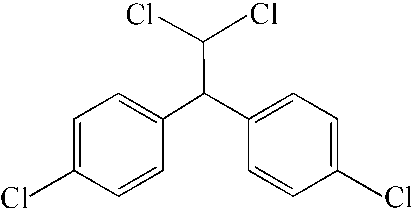 | 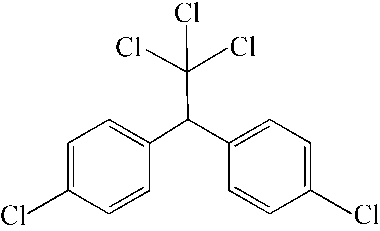 |
| *p, p’*-DDE | *p, p’*-DDD | *p, p’*-DDT |

Figure S5. The molecular structures of DDT and its metabolites (DDD and DDE).

Reference

1. National Bureau of statistics of China, *China Statistical Yearbook*. (China Statistics Press, 2013).

2. Niu, L. L. *et al.*, Status, Influences and Risk Assessment of Hexachlorocyclohexanes in Agricultural Soils Across China. *Environ. Sci. Technol.* **47** 12140 (2013).

3. Yuan, H. J. & Zhang, A. P., Determination of enantiomer fraction of selected organochlorine pesticides in soil by chiral gas chromatography (in Chinese). *Chin. Anal. Chem* **37** 630 (2009).

4. Tsilimigras M. C. B. & Fodor A. A., Compositional data analysis of the microbiome: fundamentals, tools, and challenges, annals of epidemiology (2016), doi: 10.1016/j.annepidem.2016.03.002.

5. Sun, J. *et al.*, Contamination of phthalate esters, organochlorine pesticides and polybrominated diphenyl ethers in agricultural soils from the Yangtze River Delta of China. *Sci. Total. Environ.* **544** 670 (2015).

6. Zhang, J. J., Liu, J. S., Yu, R., Liu, Q. & Wang, Y., Pollution assessment of organochlorine pesticides in urban agricultural soils of Jilin City, China. *Human and Ecological Risk Assessment: An International Journal* 10 (2015).

7. Zhang, A., Chen, Z., Ahrens, L., Liu, W. & Li, Y., Concentrations of DDTs and enantiomeric fractions of chiral DDTs in agricultural soils from Zhejiang Province, China, and correlations with total organic carbon and pH. *J. Agr. Food Chem.* **60** 8294 (2012).

8. Jiang, Y. F. *et al.*, Occurrence, distribution and possible sources of organochlorine pesticides in agricultural soil of Shanghai, China. *J. Hazard Mater.* **170** 989 (2009).

9. Yang, D. *et al.*, Residues of organochlorine pesticides (ocps) in agricultural soils of Zhangzhou City, China. *Pedosphere* **22** 178 (2012).

10. Gao, J., Zhou, H. F., Pan, G. Q., Wang, J. Z. & Chen, B. Q., Factors Influencing the Persistence of Organochlorine Pesticides in Surface Soil from the Region around the Hongze Lake, China. *Sci. Total. Environ.* **443** 7 (2013).

11. Qu, C. *et al.*, Risk assessment and influence factors of organochlorine pesticides (OCPs) in agricultural soils of the hill region: A case study from Ningde, southeast China. *J. Geochem. Explor.* **149** 43 (2015).

12. Gao, F., Jia, J. Y. & Wang, X. M., Occurrence and ordination of dichlorodiphenyltrichloroethane and hexachlorocyclohexane in agricultural soils from Guangzhou, China. *Arch. Environ. Con. Tox.* **54** 155 (2008).

13. Lv, J. G. *et al.*, Assessment of 20 organochlorine pesticides (OCPs) pollution in suburban soil in Tianjin, China. *B. Environ. Contam. Tox.* **85** 137 (2010).

14. Tao, S. *et al.*, Organochlorine pesticides in agricultural soil and vegetables from Tianjin, China. *Environ. Sci. Technol.* **39** 2494 (2005).

15. Ma, L. L., Chu, S. G. & Xu, X. B., Organic contamination in the greenhouse soils from Beijing suburbs, China. *J. Environ. Monitor.* **5** 786 (2003).

16. Shi, Y. J., Lu, Y. L., Wang, T. Y., Wang, G. & Luo, W., Comparison of organochlorine pesticides occurrence, origin, and character in agricultural and industrial soils in Beijing. *Arch. Environ. Con. Tox.* **57** 447 (2009).

17. Yu, X. M. *et al.*, Content and composition of organochlorinated pesticides in soils of central Jilin, China. *Geolog. Bull. China* **26** 1476 (2007) (in Chinese).

18. Wang, X. Q. *et al.*, Distribution and composition of organochlorine pesticides in farmland top soils of Anhui Province. **22** 3285 (2011) (in Chinese).

19. Chen, Y., Studies on HCH and DDT Pesticide Residues in Cultivated Soils in Hunan Province. *Environmental Monitoring in China* **28** 44 (2012) (in Chinese).

20. An, Q. *et al.*, Organochlorine pesticide residues in cultivated soils in the south of Jiangsu, China. *Acta Pedologica Sinica*. **41** 414 (2004) (in Chinese).

21. Yang, X. *et al.*, Dicofol application resulted in high DDTs residue in cotton fields from northern Jiangsu province, China. *J. Hazard. Mater.* **150** 92 (2008).

22. Zhou, Q. *et al.*, Distribution and sources of organochlorine pesticides in agricultural soils from central China. *Ecotox. Environ. Safe* **93** 163 (2013).

23. An, Q., Dong, Y. H., Wang, H. & Ge, C. J., Residues and distribution character of organochlorine pesticides in soils in Nanjing area. *Acta Scientiae Circumstantiae* **25** 470 (2005).

24. Li, J., Zhang, G., Qi, S., Li, X. & Peng, X., Concentrations, enantiomeric compositions, and sources of HCH, DDT and chlordane in soils from the Pearl River Delta, South China. *Sci. Total Environ.* **372** 215 (2006).

25. Velasco, A., Hernandez, S., Ramirez, M. & Ortiz, I., Detection of residual organochlorine and organophosphorus pesticides in agricultural soil in Rio Verde region of San Luis Potosi, Mexico. *J. Environ. Sci. Heal. B* **49** 498 (2014).

26. Oyekunle, J., Ogunfowokan, A. O., Torto, N. & Akanni, M. S., Determination of organochlorine pesticides in the agricultural soil of Oke-Osun farm settlement, Osogbo, Nigeria. *Environ. Monit. Assess.* **177** 51 (2011).

27. Babu, G. S. *et al.*, DDT and HCH residues in basmati rice (Oryza sativa) cultivated in Dehradun (India). *Water Air Soil Poll.* **144** 149 (2003).

28. Mishra, K., Sharma, R. C. & Kumar, S., Contamination levels and spatial distribution of organochlorine pesticides in soils from India. *Ecotox. Environ. Safe* **76** 215 (2012).

29. Covaci, A., Hura, C. & Schepens, P., Selected persistent organochlorine pollutants in Romania. *Sci. Total Environ.* **280** 143 (2001).

30. Vu, D. T., Vu, D. T., Walder, J., Schmutz, H. & Cao, T. H., Contamination by selected organochlorine pesticides (OCPs) in surface soils in Hanoi, Vietnam. *B. Environ. Contam. Tox.* **78** 195 (2007).

31. Vu, D. T., Vu, D. T., Walder, J. & Cao, T. H., Residue, Temporal Trend and Half-Life Time of Selected Organochlorine Pesticides (OCPs) in Surface Soils from Bacninh, Vietnam. *B. Environ. Contam. Tox.* **82** 516 (2009).

32. Holoubek, I. *et al.*, Soil burdens of persistent organic pollutants - Their levels, fate and risk. Part I. Variation of concentration ranges according to different soil uses and locations. *Environ. Pollut.* **157** 3207 (2009).

33. Waliszewski, S. M. *et al.*, (2008), Vol. 81, pp. 343.

34. Manz, M., Wenzel, K. D., Dietze, U. & Schuurmann, G., Persistent organic pollutants in agricultural soils of central Germany. *Sci. Total. Environ.* **277** 187 (2001).

35. Kannan, K. *et al.*, Trace organic contaminants, including toxaphene and trifluralin, in cotton field soils from Georgia and South Carolina, USA. *Arch. Environ. Con. Tox.* **45** 30 (2003).

36. Bidleman, T. F. & Leone, A. D., Soil-air exchange of organochlorine pesticides in the Southern United States. *Environ. Pollut.* **128** 49 (2004).

37. Munoz-Arnanz, J. & Jimenez, B., New DDT inputs after 30 years of prohibition in Spain. A case study in agricultural soils from south-western Spain. *Environ. Pollut.* **159** 3640 (2011).

38. Bidleman, T. F. *et al.*, Emission of legacy chlorinated pesticides from agricultural and orchard soils in British Columbia, Canada. *Environ. Toxicol. Chem.* **25** 1448 (2006).

39. Ngabe, B. & Bidleman, T. F., DDT concentrations in soils of Brazzaville, Congo. *B Environ. Contam. Tox.* **76** 697 (2006).
